# Contribution to knowledge of the genus *Chydaeus* in Xizang Autonomous Region [Tibet] and Yunnan Province, China (Coleoptera, Carabidae, Harpalini)

**DOI:** 10.3897/zookeys.171.2306

**Published:** 2012-02-24

**Authors:** Boris M. Kataev, Hongbin Liang, David H. Kavanaugh

**Affiliations:** 1 Zoological Institute, Russian Academy of Sciences, Universitetskaya nab. 1, St. Petersburg 199034, Russia; 2 Key Laboratory of Zoological Systematics and Evolution, Institute of Zoology, Chinese Academy of Sciences, Beijing 100101, China; 3 Department of Entomology, California Academy of Sciences, San Francisco, California 94118 USA

**Keywords:** Coleoptera, Carabidae, Harpalini, *Chydaeus*, China, Xizang, Yunnan, Gaoligong Shan, taxonomy, distribution, new species

## Abstract

Five new species of the genus *Chydaeus* Chaudoir, 1854 are described from China: *Chydaeus fugongensis* **sp. n.** (Shibali, Fugong County, Yunnan Province), *Chydaeus gutangensis* **sp. n.** (Gutang, Medog County, Xizang Autonomous Region [Tibet]), *Chydaeus hanmiensis* **sp. n.** (Hanmi, Medog County, Xizang Autonomous Region [Tibet]), *Chydaeus asetosus* **sp. n.** (NE of Fugong, Yunnan Province), and *Chydaeus baoshanensis* **sp. n.** (N of Baoshan, Yunnan Province). Taxonomic and faunistic notes on eleven other species occurring in Xizang and Yunnan are also provided. *Chydaeus shunichii* Ito, 2006 is re-described, based on specimens from Lushui County, Yunnan. *Chydaeus kumei* Ito, 1992 is treated as a subspecies of *Chydaeus andrewesi* Schauberger, 1932 [NEW STATUS**]**. The taxonomic status of *Chydaeus guangxiensis* Ito, 2006 is discussed. The following taxa are recorded from China for the first time: *Chydaeus obtusicollis* Schauberger, 1932 (Xizang and Yunnan), *Chydaeus malaisei* Kataev & Schmidt, 2006 (Yunnan), *Chydaeus semenowi* (Tschitschérine, 1899) (Xizang and Yunnan), *Chydaeus andrewesi andrewesi* Schauberger, 1932 (Xizang and Yunnan), *Chydaeus andrewesi kumei* Ito (Yunnan), *Chydaeus bedeli interjectus* Kataev & Schmidt, 2002 (Xizang), and *Chydaeus bedeli vietnamensis* Kataev & Schmidt, 2002 (Yunnan).

## Introduction

Genus *Chydaeus* Chaudoir, 1854 is a moderately diverse group of anisodactyline species, members of which are distinctly recognizable in having the dentate mentum completely fused with the submentum. The genus includes more than 40 species distributed mainly over mountainous regions of southeastern Asia, from the Himalayas and China to the Sunda Isles; however, three species are known from New Guinea (Baehr 2007) and one from Australia (Baehr 2004). Many species of *Chydaeus* are apterous and have very restricted geographical distributions. Some species, including ones with both fully-winged and brachypterous adults, demonstrate considerable geographical variation in their morphology, and several of them are treated as polytypic, with between two and five subspecies each. No comprehensive revision of this genus has been published since Schauberger’s work (1934b) dealing with the 11 species known at that time. *Chydaeus* species occurring in the Himalayan region were studied by Kataev and Schmidt (2002, 2006), who assigned these species to several species groups based mainly on the male genitalic structure and metacoxal and elytral chaetotaxy. The *Chydaeus* fauna of China is still inadequately known. Thirteen species have been described or recorded from China (three of them only from Taiwan), but many species in the region remain undescribed.

The present paper provides descriptions for five new species from Xizang Autonomous Region (Tibet) and Yunnan Province, as well as taxonomic and faunistic notes on some previously described species also occurring in this region. These species belong to seven species groups, two of them (the *kasaharai* and *gutangensis* groups) are established in this paper. The order of presentation of species treatments here follows that of Kataev and Schmidt (2002, 2006), with new species arranged according to likely species group relationships. Because additional new species of *Chydaeus* undoubtedly remain to discovered and described from inadequately sampled portions of the study area, presentation of a key to species would be of dubious value at this time.

## Material and methods

As in our preceding papers (Kataev and Liang 2004, 2005, 2007), this study is based mainly on examination of the collections of the Institute of Zoology, Chinese Academy of Sciences, Beijing, but also on material collected during the period 1998–2007 through a joint project of the Institute of Zoology (Beijing) and the Kunming Institute of Zoology (Yunnan) of the Chinese Academy of Sciences and the California Academy of Sciences (San Francisco) for a biodiversity inventory of the Gaoligong Shan (Mountains) of western Yunnan Province.

For the present study, we examined a total of 1,666 specimens of *Chydaeus* species. The following abbreviations are used for the depositories of the specimens examined:

CAS California Academy of Sciences, San Francisco, U.S.A

cBL&KB Coll. I. Belousov & I. Kabak (St. Petersburg, Russia)

cFED Coll. D. Fedorenko (Moscow, Russia)

cSCH Coll. J. Schmidt (Marburg, Germany)

cWR Coll. D.W. Wrase (Berlin, Germany)

IOZ Institute of Zoology, Chinese Academy of Sciences, Beijing, China

ZIN Zoological Institute of the Russian Academy of Sciences, St. Petersburg, Russia.

Measurements were taken as follows: body length, measured from the anterior margin of the clypeus to the elytral apex; width of head, measured as the maximum linear distance across the head, including the compound eyes (HWmax), and as the minimum linear distance across the neck constriction just behind the eyes (HWmin); length of pronotum (PL), measured along its median line; length of elytra (EL), measured from the basal border in the scutellar region to the apex of the sutural angle; maximum width of pronotum (PWmax) and of elytra (EW), both measured at their broadest point; minimum width of pronotum (PWmin), measured at its narrowest point near the hind angles; length and width of metepisterna, measured along their inner and anterior margins, respectively.

## Taxonomy

### Chydaeus shunichii

Ito, 2006

http://species-id.net/wiki/Chydaeus_shunichii

Figs 1–9 56 64–65

#### Material examined.

 A total of 15 specimens (6 males and 9 females, including 2 males and 5 females in CAS, 3 males and 4 females in IOZ, and 1 male in ZIN) were examined from the following localities: **China**, **Yunnan Province**. *Lushui County*: 1 male, Gaoligongshan, Nujiang Pref., Pianma Yakou, 3200 m, 25°54.4'N, 98°41.0'E, 11.X.1998, Stop 98-113A, D.H. Kavanaugh, C.E. Griswold, C. Ferraris & C.-L. Long leg. (IOZ); 3 females, Pianma, Fengxue Yakou, roadside, 25.97244°N, 98.68376°E, 3150 m, 11.V.2005, H.B. Liang leg. (CAS, IOZ); 3 males, 4 females, Luzhang, Fengxue Yakou, roadside, 25.97347°N, 98.68780°E, 3130 m, 17.V.2005, D. Kavanaugh & D.Z. Dong leg. (CAS, IOZ, ZIN); 1 female, same data, but 25.97360°N, 98.68905°E, 3120 m, 17.V.2005, H.B. Liang leg. (IOZ); 1 female, same data, but Y.H. San leg. (IOZ); 1 male, Luzhang, Fengxue Yakou, road, 25.97410°N, 98.67716°E, 3120 m, 18.V.2005, D. Kavanaugh & D.Z. Dong leg. (CAS); 1 male, Luzhang, Yaojiaping, roadside, 25.97526°N, 98.71000°E, 2513 m, 19.V.2005, H.B. Liang leg. (IOZ).

#### Re-description

**(**based on the specimens from Lushui County, Yunnan). Size: Body length 6.9–8.0 mm, width 3.5–3.9 mm.

Color: Body black, slightly shiny on dorsum, labrum and bases of mandibles paler, reddish brown in some specimens. Antennae brown, with antennomere 1 more or less infuscated. Palpi yellowish brown. Legs dark brown to black, with tibiae basally and tarsi brown.

Microsculpture: Head with dorsal microsculpture visible throughout, comprised of very fine, more or less isodiametric meshes. Pronotum with microsculpture clearly visible throughout, comprised of distinct isodiametric meshes. Elytra with microsculpture developed throughout, comprised of distinct, slightly transverse meshes.

Head: Comparatively large (HWmax/PWmax = 0.69–0.72 and HWmin/PWmax = 0.59-0.61), impunctate, with small and moderately convex eyes; tempora flat, sloped to neck. Clypeus slightly concave and with slightly convex bead along anterior margin. Frontal suture distinct, slightly impressed. Frontal foveae small, faintly deepened; clypeo-ocular prolongations absent. Supraorbital setae situated slightly behind hind margins of eyes. . Antennae short, not extended to pronotal basal margin, with antennomeres 5 to 7 each 1.4–1.6 times as long as wide. Labrum deeply emarginate apically. Left mandible truncate at apex. Ligular sclerite more or less parallel-sided, with apex straight and apical angles slightly extended laterad.

**Figures 1–9.**
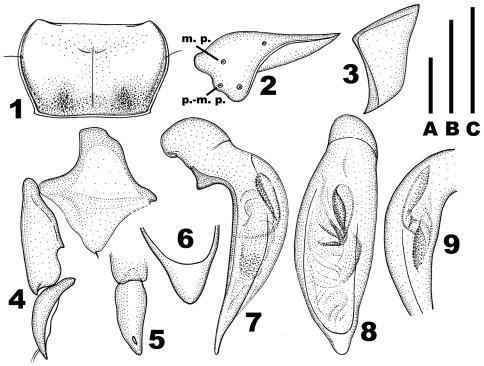
*Chydaeus shunichii* Ito (Pianma Yakou). **1** Pronotum **2** Left metacoxa (m. p. = medial setigerous pore, p.-m. p. = postero-medial setigerous pore) **3** Left metepisternum. **4** Right hemisternite and stylus, ventral view **5** Stylus, lateral view **6** Terminal lamella of median lobe, dorsal view **7** Median lobe, left lateral view **8** Median lobe, dorsal view **9** Middle portion of median lobe, right lateral view. Scale lines: **A** = 1.0 mm (Fig. 1), **B** = 0.5 mm (Fig. 6), **C** = 1.0 mm (Figs 2–5, 7–9).

Pronotum (Fig. 1): Markedly transverse (PWmax/PL = 1.55–1.63), narrowed basad (PWmax/PWmin =1.22–1.30) and widest in anterior third. Sides distinctly rounded in anterior two-thirds and very slightly rounded, almost rectilinearly convergent basad in posterior third, with one lateral seta on each side in apical third. Apical margin arcuately emarginate, bordered only laterally. Basal margin nearly straight, slightly oblique at basal angles, distinctly bordered throughout, slightly wider than apical margin and slightly shorter than elytral base between humeral angles. Apical angles slightly less than 90°, produced anteriad and narrowly rounded at apices. Basal angles obtuse, sharp at apices, each with a small, obtuse denticle extended laterad. Pronotal disc moderately convex, slightly depressed basally, slightly sloped to sides and more abruptly sloped to apical angles. Lateral depressions present as narrow furrows behind apical angles, slightly widened behind lateral setigerous pore, and either extended basad to pronotal base or fused in basal quarter with large latero-basal depressions. Basal foveae distinct within latero-basal depressions, comparatively small, somewhat flat or slightly deepened. Areas between basal foveae and between basal foveae and lateral depressions slightly convex in most specimens. Pronotal surface coarsely and irregularly punctate along base and in lateral depressions, finer and sparser punctation present also along apical margin.

Elytra: Oval, markedly rounded at sides, wide (EL/EW= 1.20–1.29, EL/PL = 2.58–2.69, EW/PWmax = 1.32–1.37), widest approximately at middle, not fused along suture. Humeri widely rounded at apices, each with indistinct denticle hardly visible from behind; sides just behind humeral angles markedly rounded. Subapical sinuations present, shallow. Sutural angles not separated from each other, less than 90°, with apices blunt in both sexes. Basal borders faintly sinuate, joined with lateral margin at a markedly obtuse angle. Striae smooth, impunctate, faintly impressed along entire length. Parascutellar striae present, short, basal setigerous pores present; in some specimens, distal part of parascutellar stria connected with first (sutural) stria. Intervals faintly convex along entire length, impunctate, only slightly narrowed toward apices. Umbilicate setal series widely interrupted at middle, with anterior group comprised of 7 or 8 setigerous pores and posterior group comprised of 8 or 9 such pores.

Hindwings: Reduced to small scales.

Venter: Prosternum with scattered and short setae apically. Prosternal medial process not projected posteriad. Proepisterna smooth, impunctate. Metepisterna (Fig. 3) distinctly narrowed posteriad, approximately as wide as long. Sternum VII (anal) in both sexes with two pairs of setae along apical margin and widely rounded at apex. Tergum VII (anal) of female rounded at apex.

Legs: Metacoxae (Fig. 2) with additional postero-medial setigerous pore and, in most specimens, with additional medial setigerous pore at least on one side. Metafemora with two setae along posterior margin. Protibiae with one ventroapical spine, the outer margin in both sexes with three or four uniform preapical spines. Tarsi glabrous dorsally, at most with a few setae on enlarged male protarsomeres; tarsomere 5 with three pairs of lateroventral setae. Metatarsi in both sexes notably shorter than minimum linear distance across neck constriction just behind eyes; tarsomere 1 longer than tarsomere 2 and shorter than tarsomeres 2+3. In males, protarsi moderately enlarged, with tarsomere 1 about as long as wide, tarsomeres 2 to 4 distinctly wider than long and tarsomeres 1 to 4 with adhesive vestiture ventrally (only apically on tarsomere 1); mesotarsi comparatively slightly enlarged, with tarsomere 1 slightly longer than wide, tarsomere 2 approximately as long as wide, tarsomere 3 slightly wider than long, tarsomere 4 much smaller than tarsomeres 2 and 3 and deeply concave apically; mesotarsomeres 2 and 3 with adhesive vestiture ventrally.

Female genitalia (Figs 4–5): Apical stylomere comparatively slightly curved.

Aedeagus (Figs 6–9): Median lobe markedly widened medially (dorsal aspect), markedly bent ventrad behind basal bulb and with terminal lamella slightly curved dorsad (lateral aspect), its ventral margin nearly straight medially. Terminal lamella (Fig. 6) flat, triangular in dorsal aspect, slightly wider than long, narrowly rounded at apex and without any apical capitulum. Apical orifice in dorsal position, prolonged to basal bulb. Internal sac with several spiny patches.

#### Distribution.

Fig. 56. This species is known only from the Gaoligong Shan in Lushui and Tengchong counties, western Yunnan Province, China, at elevations of 2500–3200 m.

#### Habitat.

 Specimens were collected in roadside and road cut open areas, hidden under stones and other debris during daylight hours and active on the soil surface at night (Figs 64–65).

#### Remarks.

*Chydaeus shunichii* was originally described on the basis of one male from “Dakei, alt. 2430–2440 m, Mts. Gaoligongshan, Tengchong Xian, Yunnan” (Ito 2006). The specimens from Lushui County share with the holotype most of the characters listed by Ito in the original description, but they differ slightly in the male genitalia. The apical part of the median lobe is less markedly curved dorsad than that illustrated by Ito (2006) and with a more acute, unbordered apex. The ventral side of the median lobe is nearly straight medially in lateral aspect, and the internal sac possesses the very characteristic spiny armature, without “a peg-shaped sclerite” mentioned by Ito (2006) for the holotype. In addition, there are also differences in several characters of external morphology. In the specimens from Lushui County, the head is smooth (“sparsely and coarsely punctate and transverse rugosities on frons” in the holotype), the pronotal dorsal microsculpture consists of the isodiametric meshes (“consisting of transverse meshes” in the holotype), the elytra have seven to eight setigerous pores in anterior group of the umbilicate setal series (five in the holotype), and only mesotarsomeres 2 and 3 in males have adhesive vestiture ventrally (“mid tarsi of male bearing spongy adhesive hairs on ventral surface of 2nd to 4th segments” in the holotype). One of us [HBL] was able to examine the holotype of *Chydaeus shunichii* at Ito’s home (Kawanishi City, Japan) and found that at least the aedeagus of the holotype was described and illustrated inaccurately by Ito (2006) because it is identical to that of the specimens from Lushui County as described above. The extent to which specimens of *Chydaeus shunichii* from Lushui County actually differ morphologically from those from Tengchong County requires further study based on additional material from Tengchong.

*Chydaeus shunichii* members are similar in habitus and male genitalia to those of *Chydaeus kasaharai* Ito, 2002, which was described from Dashennongjia Mountain in the eastern part of the Daba Shan (western Hubei Province). However, in members of the latter species, the head and elytra are punctate at least laterally, the basal borders of the elytra are markedly sinuate laterally and form with lateral margin of elytra a sharp, nearly rectangular angle, the elytral striae are distinctly crenulate, the tarsi are pubescent dorsally, and the median lobe of the aedeagus is narrower, with much longer terminal lamella and with only one narrow, curved spiny patch in the internal sac apically. These two species (*Chydaeus shunichii* and *Chydaeus kasaharai*) are distinct from other known species of *Chydaeus* in their morphology and, in our opinion, form a natural species group named here the *kasaharai* group. The main distinctive characters of members of this group are: the wide body, the elytra with comparatively short parascutellar stria and with basal parascutellar setigerous pore present, the umbilicate setal series of elytra usually with a more or less wide gap medially, rarely continuous, the metepisterna slightly wider than long or approximately as wide as long, and the metacoxae with a posterolateral setigerous pore and (in most specimens) additional medial setigerous pores. Members of the *kasaharai* group are very similar in these characters to those of the *irvinei* group (sensu Kataev and Schmidt 2002, 2006), but the latter are distinguished from members of the *kasaharai* group in having elytra without a parascutellar setigerous pore and without a parascutellar striole (the latter present as only a rudiment in a few specimens).

### Chydaeus fugongensis

Kataev & Kavanaugh sp. n.

urn:lsid:zoobank.org:act:E9FE9140-4A0A-4A14-AADB-EF636C7CE0F6

http://species-id.net/wiki/Chydaeus_fugongensis

Figs 10–15 51 56 66

#### Type material.

 Holotype, a male, deposited in IOZ, labeled: “China, Yunnan, Fugong Co., Lishadi town, Shibali, 6 km up, roadside, 27.17628°N, 98.74167°E, 2920 m, 2.V.2004, H.B. Liang & X.Y. Li leg”.

#### Description

 (male holotype only). Dorsal habitus as in Fig. 51. Size: Body length 8.2 mm, width 3.8 mm.

Color: Body black, shiny on dorsum; mandibles basally, pronotum at basal angles and elytral epipleura slightly paler, reddish black. Antennae brown, with antennomeres 3 and 4 infuscated. Palpi light brown. Tarsi dark brown.

Microsculpture: Head with dorsal microsculpture comprised of fine isodiametric meshes, visible throughout, except effaced on frons and vertex. Pronotum with microsculpture visible throughout, comprised of fine isodiametric meshes basally and laterally and of slightly transverse, nearly effaced meshes on remaining surface. Elytra with microsculpture distinct throughout, comprised of fine, slightly transverse meshes.

Head: Comparatively large (HWmax/PWmax = 0.73 and HWmin/PWmax = 0.63), covered with micropunctures and very fine wrinkles; tempora slightly convex, almost flat, sloped to neck. Clypeus slightly concave and with slightly convex bead apically. Frontal suture distinct, slightly impressed. Frontal foveae small, oval, slightly deepened; clypeo-ocular prolongations absent. Supraorbital setae situated just behind hind margins of eyes. Eyes small and moderately convex. Antennae slightly extended beyond pronotal basal margin, with antennomeres 5 to 7 each about 1.6 times as long as wide. Labrum distinctly emarginate apically. Left mandible truncate at apex. Ligular sclerite nearly parallel-sided, straight at apex.

**Figures 10–15.**
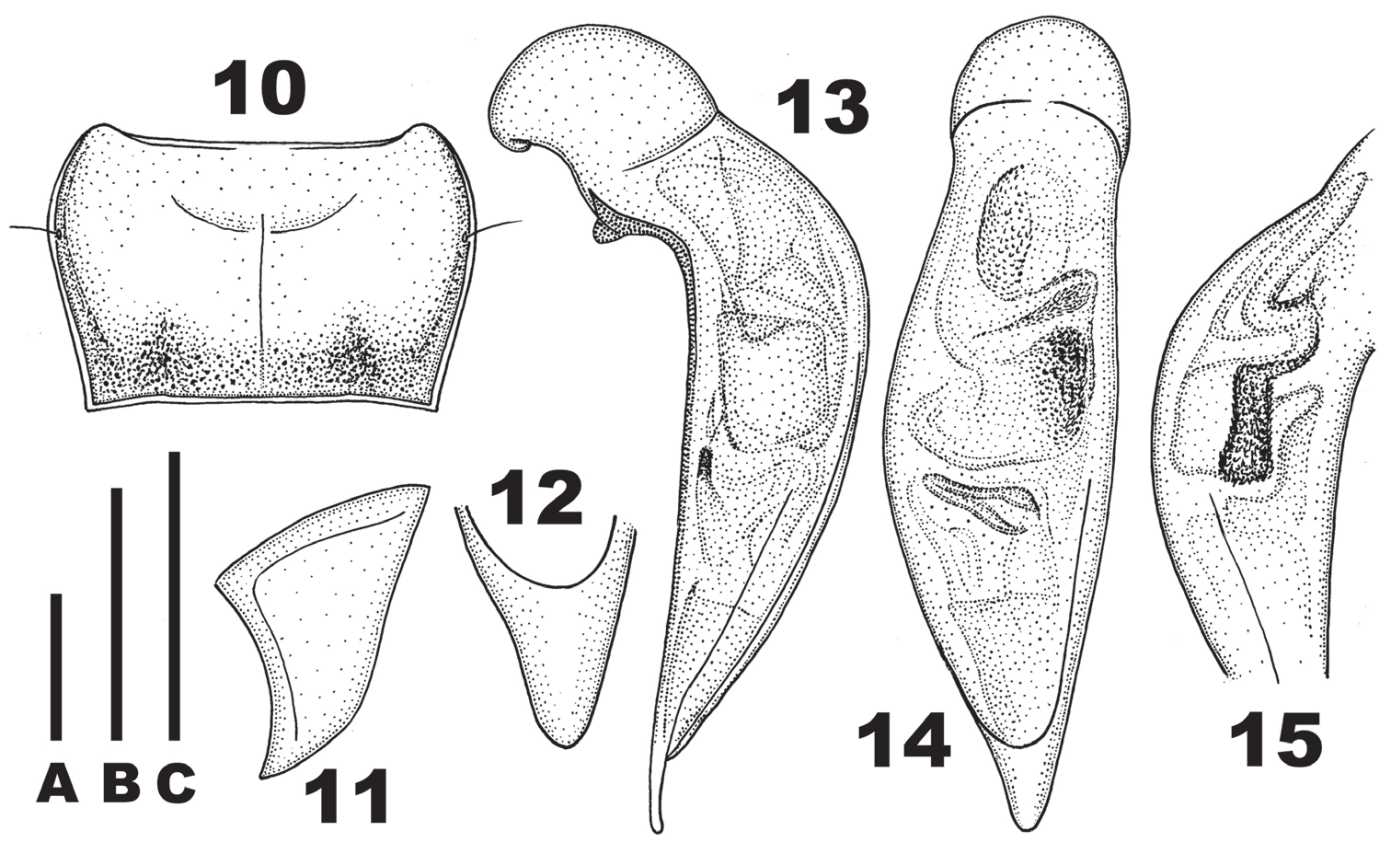
*Chydaeus fugongensis* sp. n. (holotype) **10** Pronotum **11** Left metepisternum **12** Terminal lamella of median lobe, dorsal view **13** Median lobe, left lateral view **14** Median lobe, dorsal view **15** Middle portion of median lobe, right lateral view. Scale lines: A = 1.0 mm (Fig. 10), B = 0.5 mm (Fig. 12), C = 1.0 mm (Figs 11, 13–15).

Pronotum (Fig. 10): Markedly transverse (PWmax/PL = 1.64), narrowed basad (PWmax/PWmin = 1.26) and widest before middle. Sides rounded in anterior half and very faintly sinuate in basal half, with one lateral setigerous pore on each side before middle. Apical margin arcuately emarginate, bordered only laterally. Basal margin very broadly emarginate, almost straight, distinctly bordered throughout, slightly wider than apical margin and slightly narrower than elytral base between humeral angles. Apical angles slightly less than 90°, extended anteriad and narrowly rounded at apices. Basal angles slightly more than 90°, sharp at apices, each with a tiny denticle produced laterad. Pronotal disc moderately convex, slightly depressed basally, slightly sloped to sides and more abruptly sloped to apical angles. Lateral depressions present as narrow furrows behind apical angles, slightly widened behind lateral setigerous pore, and fused in basal quarter with large laterobasal depressions. Basal foveae elongate, moderately deep. Areas between basal foveae and between basal foveae and lateral depressions slightly convex. Pronotal surface coarsely and irregularly punctate along base and in lateral depressions, finer and sparser punctation present along apical margin and in central portion of disc.

Elytra: Oval, rounded at sides, moderately wide (EL/EW= 1.34, EL/PL = 2.83, EW/PWmax = 1.29), widest approximately at middle, not fused along suture. Humeri slightly prominent, widely rounded at apices, each with indistinct denticle visible only from behind; sides just behind humeral angles markedly rounded. Subapical sinuations shallow. Sutural angles not separated from each other, less than 90°, blunt at apices. Basal borders slightly sinuate, joined with lateral margin at very obtuse angle. Striae impunctate, faintly impressed along entire length. Parascutellar striae present, short, with basal setigerous pores present. Intervals slightly convex, almost flat, impunctate. Umbilicate setal series widely interrupted at middle, with anterior group comprised of six setigerous pores and posterior group comprised of seven setigerous pores on right side and of nine setigerous pores on left side.

Hindwings: Reduced to small scales.

Venter: Prosternum covered with scattered and very short setae. Prosternal medial process not projected posteriad. Proepisterna smooth, impunctate. Metepisterna (Fig. 11) distinctly narrowed posteriad, slightly wider than long. Sternum VII (anal) in male with two pairs of setae along apical margin and slightly truncate at apex.

Legs: Metacoxae with additional posteromedial setigerous pore and with one or two additional medial setigerous pores. Metafemora with two setae along posterior margin. Protibiae with one ventroapical spine, the outer margin with one stouter spine and three slenderer spines apically. Tarsi glabrous dorsally, tarsomere 5 with three or four pairs of lateroventral setae. Metatarsi approximately equal to minimum linear distance across neck constriction just behind eyes; tarsomere 1 distinctly longer than tarsomere 2 and slightly shorter than tarsomeres 2+3. Protarsi (in male) moderately enlarged, with tarsomere 1 slightly longer than wide, tarsomeres 2 slightly wider than long, tarsomeres 3 and 4 distinctly wider than long and tarsomeres 1 to 4 with adhesive vestiture ventrally (only apically on tarsomere 1); mesotarsi comparatively slightly enlarged, with tarsomere 1 (not enlarged) distinctly longer than wide, tarsomere 2 slightly longer than wide, tarsomere 3 slightly wider than long, tarsomere 4 much smaller than tarsomeres 2 and 3 and deeply concave apically; mesotarsomeres 2 and 3 with adhesive vestiture ventrally.

Aedeagus (Figs 12–15): Median lobe moderately widened medially (dorsal aspect), bent ventrad behind basal bulb and with terminal lamella slightly curved dorsad (lateral aspect) and with ventral margin nearly straight medially. Terminal lamella (Fig. 12) flat, in dorsal aspect triangular, slightly longer than wide, narrowly rounded at apex and without any apical capitulum. Apical orifice in dorsal position, prolonged to basal bulb. Internal sac with several spiny patches.

#### Distribution.

Fig. 56. This new species is known only from the type locality (Shibali, Lishadi town, Fugong County) in the northern part of Gaoligong Shan, northwestern Yunnan Province, China.

#### Habitat.

 The holotype specimen was collected in a roadside open area hidden under a stone (Fig. 66).

#### Specific epithet.

 The specific epithet refers to Fugong, the county in Yunnan Province, where the type specimen was collected.

#### Remarks.

 Like *Chydaeus shunichii* re-described above, *Chydaeus fugongensis* belongs to the *kasaharai* group because the holotype specimen has all the distinctive features of that group, namely: elytra with comparatively short parascutellar striae and with basal setigerous pores, the umbilicate setal series with wide gap medially, metepisterna slightly wider than long, and metacoxae with a posterolateral setigerous pore and additional medial setigerous pores. Within this group, the single known *Chydaeus fugongensis* male is most similar to those of *Chydaeus shunichii*, differing from them in the following characters: body, particularly the elytra, more elongate, pronotal sides slightly sinuate in basal half, pronotal microsculpture comprised of fine isodiametric meshes basally and laterally and of slightly transverse and nearly effaced meshes on the remaining surface, sternum VII (anal) slightly truncate at apex, median lobe of the aedeagus narrower, less markedly curved ventrad and with a longer terminal lamella, and the internal sac with characteristic armature. The *Chydaeus fugongensis* male is similar to those *Chydaeus kasaharai* in the shape of the median lobe, but differs from them in having impunctate elytra with much wider inner humeral angles (formed by the junction of the basal border and lateral elytral margin) and dorsally glabrous tarsi, and in the structure of the internal sac of the aedeagus. In addition, the basal bulb of the median lobe in *Chydaeus fugongensis* is relatively smaller than that in *Chydaeus kasaharai* males.

### Chydaeus gutangensis

Kataev & Liang sp. n.

urn:lsid:zoobank.org:act:D7C76382-D513-47C5-8240-D09F599D2965

http://species-id.net/wiki/Chydaeus_gutangensis

Figs 16–22 52 56

#### Type material.

 Holotype, a male, deposited in IOZ, labeled: “**China**, Xizang Autonomous Region, Medog Co., Gutang Township, 2000 m, 16.X.1982, Han Yinheng leg.”

#### Description

 (male). Dorsal habitus as in Fig. 52. Size: Body length 9.3 mm, width 4.0 mm.

Color: Body black, slightly shiny on dorsum, almost matte due to fine micropunctation and distinct, granulate microsculpture; two penultimate abdominal sterna V and VI along posterior margin and sternum VII (anal) in apical portion clearly paler, light brown. Antennae, palpi, and legs light brown, femora slightly infuscated.

Microsculpture: Head with dorsal microsculpture distinct throughout, comprised of very distinct, isodiametric, almost granulate meshes. Pronotum with microsculpture distinct throughout, comprised of distinct isodiametric and slightly transverse meshes. Elytra with microsculpture distinct throughout, comprised of isodiametric meshes, granulate on three lateral intervals.

Head: Comparatively large (HWmax/PWmax = 0.70 and HWmin/PWmax = 0.59), covered with micropunctures and very fine wrinkles on dorsum; tempora short, slightly convex. Clypeus slightly depressed laterally and apically, with apical margin slightly concave and unbordered. Frontal suture superficial. Frontal foveae moderately wide and shallow, clypeo-ocular prolongations short and faintly impressed. Supraorbital setae situated at level of hind margins of eyes. Eyes slightly convex. Antennae short [apical antennomeres missing], with antennomeres 5 to 7 each about 1.8–2.0 times as long as wide. Labrum distinctly emarginate apically. Left mandible blunt at apex. Apex of ligular sclerite concave, with apical angles slightly projected laterally.

**Figures 16–22.**
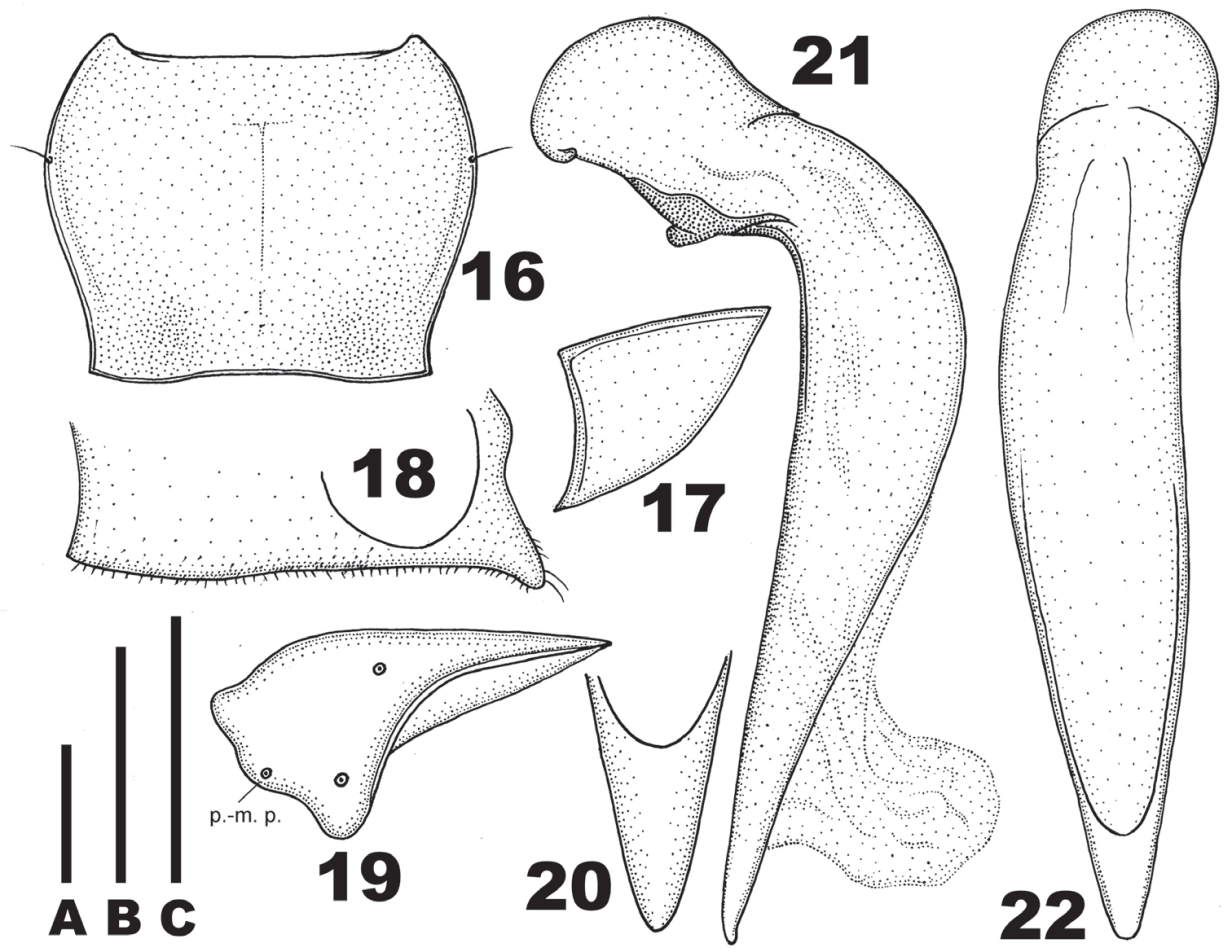
*Chydaeus gutangensis* sp. n. (holotype) **16** Pronotum **17** Left metepisternum **18** Ventral side of prosternum, right lateral view **19** Left metacoxa (p.-m. p. = postero-medial setigerous pore) **20** Terminal lamella of median lobe, dorsal view **21** Median lobe, left lateral view. **22** Median lobe, dorsal view. Scale lines: **A** = 1.0 mm (Fig. 16), **B** = 0.5 mm (Fig. 20), **C** = 1.0 mm (Figs 17–19, 21–22).

Pronotum (Fig. 16): Relatively long (PWmax/PL = 1.36), distinctly narrowed basad (PWmax/PWmin =1.27) and widest in anterior third. Sides gently rounded anteriorly, moderately sinuate in basal half and slightly divergent just before base; each side with one lateral setigerous pore in anterior third. Apical margin almost straight, bordered only near apical angles. Basal margin slightly emarginate medially, slightly oblique laterally, faintly bordered throughout, very slightly wider than apical margin and slightly narrower than elytral base between humeral angles. Apical angles acute, distinctly but not markedly produced anteriad. Basal angles almost 90°, with sharp apices, not denticulate. Pronotal disc moderately convex, not depressed basally, moderately sloped to sides and abruptly sloped to apical angles. Lateral depressions narrow anteriorly, slightly widened behind middle and indistinct at basal angles. Basal foveae wide and very shallow. Area at basal angles flat. Pronotal surface throughout densely micropunctate and covered with very fine wrinkles; laterobasal areas slightly more coarsely punctate.

Elytra: Oval, gradually rounded at sides, moderately wide (EL/EW= 1.40, EL/PL = 2.38, EW/PWmax = 1.25), widest at middle and fused along suture. Humeri rounded, without denticles at apices. Subapical sinuations very shallow. Sutural angles acute, sharp at apices, not separated from each other. Basal borders markedly sinuate, joined with lateral margin at an acute angle. Striae superficial, very fine, impunctate, slightly crenulate; inner striae nearly effaced at apex. Parascutellar striae rudimentary, each with a basal setigerous pore. Intervals absolutely flat, impunctate. Umbilicate setal series without distinct gap medially.

Hindwings: Reduced to small scales.

Venter: Prosternum finely punctate and pubescent. Medial prosternal process prominent and projected posteriad (Fig. 18). Proepisterna smooth. Metepisterna (Fig. 17) distinctly wider than long and slightly narrowed posteriad. Sternum VII (anal) with two pairs of setae along apical margin, rounded at apex.

Legs: Metacoxae (Fig. 19) with posteromedial setigerous pore and without additional, setigerous or non-setigerous foveae medially. Metafemora with two setae along posterior margin. Protibiae with one preapical spine at outer distal margin and one ventroapical spine. Tarsi distinctly setose dorsally, tarsomere 5 with three pairs of lateroventral setae. Metatarsi slender, slightly longer than maximum width of head, with tarsomere 1 much longer than tarsomere 2, but distinctly shorter than tarsomeres 2+3. Male protarsi markedly enlarged (tarsomeres 2 and 3 much wider than long and tarsomeres 1 to 4 with adhesive vestiture ventrally); mesotarsi moderately enlarged (tarsomere 1 narrow, distinctly longer than wide and with only a pair of adhesive scales apically; tarsomere 2 about 1.2 times as long as wide, with adhesive vestiture ventrally; tarsomere 3 approximately as long as wide, with adhesive vestiture ventrally; and tarsomere 4 small, deeply concave apically and without adhesive vestiture ventrally).

Aedeagus (Figs 20–22): Median lobe symmetrical, comparatively slender, markedly bent ventrad just behind basal bulb and with straight ventral margin. Sides rounded in middle portion and convergent in apical portion. Terminal lamella (Fig. 20) about 1.5 times as long as wide, triangular, narrowly rounded at apex, without apical capitulum. Apical orifice in dorsal position, prolonged to basal bulb. Internal sac without distinct sclerotic elements.

#### Distribution.

Fig. 56. This new species is known only from the type locality (Gutang Township) in the eastern Himalaya, the southeastern part of Xizang Autonomous region (Tibet), Medog County, China.

#### Specific epithet.

 The specific epithet refers to Gutang in Xizang Autonomous Region, where the type specimen was collected.

#### Remarks.

 This new species is isolated taxonomically from other known species of the genus and we include it in separate species group (the *gutangensis* group). Its members may be recognized by the following combination of characters: medial prosternal process distinctly projected posteriad, elytra with parascutellar strioles rudimentary and basal setigerous pores present, inner striae nearly effaced at apex, the umbilicate setal series without a gap at middle, metepisterna distinctly wider than long, slightly narrowed posteriad, metacoxae with a posteromedial setigerous pore and without additional setigerous or nonsetigerous foveae medially, and tarsi distinctly setose dorsally. The prosternum with the medial process distinctly projected posteriad is unique among all species of *Chydaeus* known to us. In adults of all other species, the medial prosternal process is either not or only slightly projected posteriad (as in *Chydaeus hanmiensis* described below).

The single known male of *Chydaeus gutangensis* is most similar to members of the *irvinei* group species (sensu Kataev and Schmidt 2002, 2006), which have metacoxae with an additional posteromedial setigerous pore, wide metepisterna and rudimentary parascutellar strioles, but is distinguished from them by the presence of the parascutellar setigerous pore on the elytra, dorsally setose tarsi, and greater body size (body length less than 8.0 mm in species of the *irvinei* group) among other features. Like members of some species of the *irvinei* group, the elytra of the holotype of *Chydaeus gutangensis* are fused along the suture.

The holotype male of *Chydaeus gutangensis* is similar also to the members of the *kasaharai* group in having wide metepisterna and additional posteromedial setigerous pores on the metacoxae, however it differs from them in having the body more elongate, the medial prosternal process distinctly projected posteriad, and the elytra with almost completely reduced parascutellar strioles, very fine striae that are effaced apically, and the umbilicate setal series more uniformly spaced, without a distinct gap near the middle.

### Chydaeus hanmiensis

Kataev & Liang sp. n.

urn:lsid:zoobank.org:act:DC93417A-362E-4AD4-8D37-2EA70DF3508E

http://species-id.net/wiki/Chydaeus_hanmiensis

Figs 23–29 53 56

#### Type material.

 Holotype, a male, deposited in IOZ, labeled: “China, Xizang Autonomous Region, Medog Co., Baibung town, Hanmi, 2200 m, 14.VIII.2005, Tang Liang leg.”

#### Description

 (male). Dorsal habitus as in Fig. 53. Size: Body length 8.7 mm, width 3.8 mm.

Color: Body dark piceous; antennae brown, palpi slightly lighter brown; femora black, tibiae dark brown, tarsi brown.

Microsculpture: Head with dorsal microsculpture very fine, distinct throughout, comprised of more or less isodiametric meshes. Pronotum with microsculpture comprised of distinct isodiametric and slighty transverse meshes, except effaced on disc. Elytra with microsculpture very fine, comprised of slightly transverse meshes on disc and of isodiametric meshes on two lateral intervals.

Head: Comparatively large (HWmax/PWmax = 0.73 and HWmin/PWmax = 0.62), very finely micropunctate on dorsum and covered with fine wrinkles mainly in and around frontal foveae; tempora moderately long, slightly convex. Apex of clypeus concave, very faintly bordered. Frontal suture thin, faintly impressed, clypeo-ocular prolongations thin, not deepened, extended to supraorbital furrows. Supraorbital seta situated at level of hind margin of eye. Eyes moderately convex. Antennae short, not extended to pronotal basal margin, with antennomeres 5 to 7 each about 1.4–1.5 times as long as wide. Labrum distinctly emarginate apically. Left mandible truncate at apex. Ligular sclerite slightly widened anteriorly, with straight apex.

**Figures 23–29.**
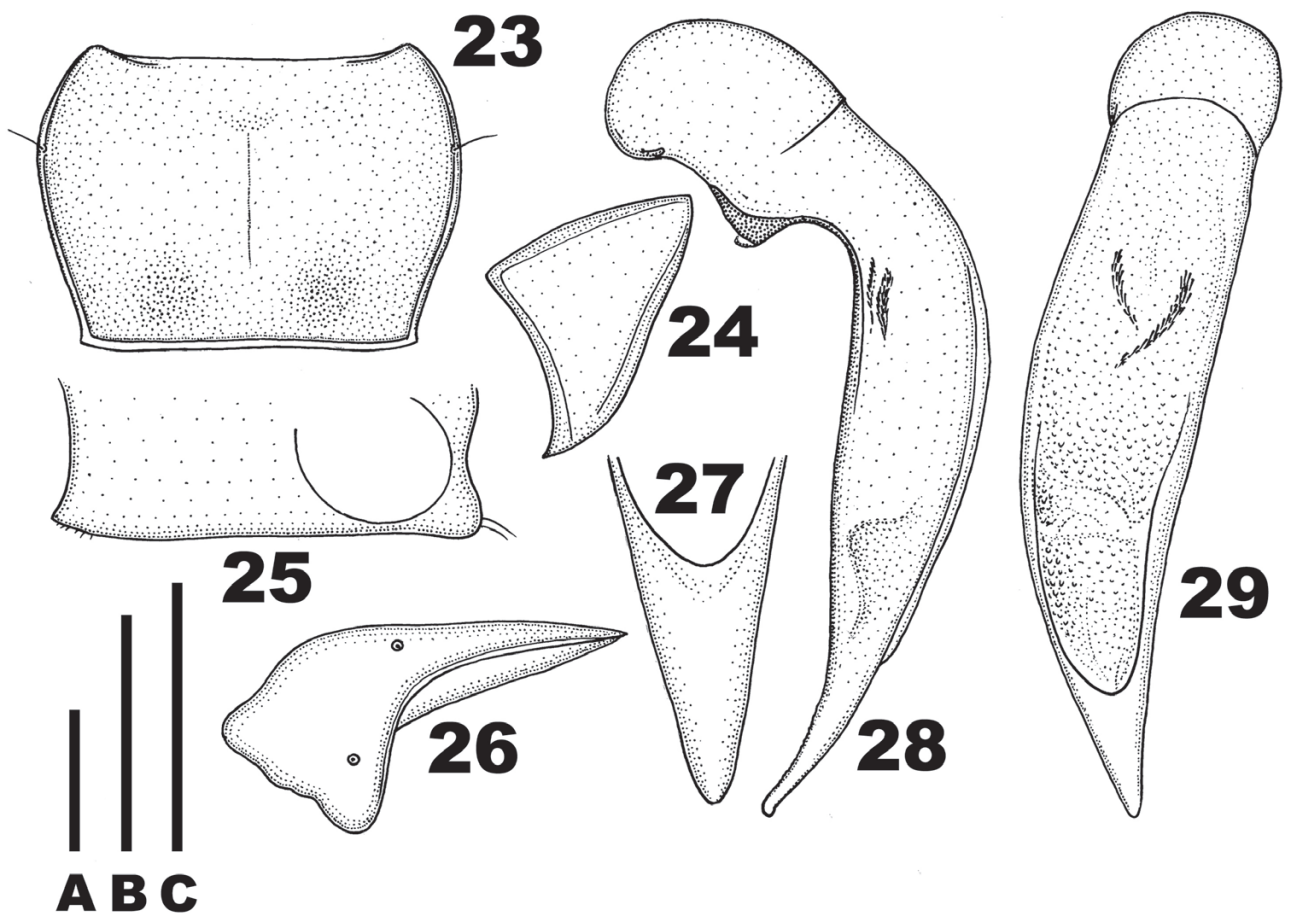
*Chydaeus hanmiensis* sp. n. (holotype) **23** Pronotum **24** Left metepisternum **25** Ventral side of prosternum, right lateral view **26** Left metacoxa **27** Terminal lamella of median lobe, dorsal view **28** Median lobe, left lateral view **29** Median lobe, dorsal view. Scale lines: **A** = 1.0 mm (Fig. 23), **B** = 0.5 mm (Fig. 27), **C** = 1.0 mm (Figs 24–26, 28, 29).

Pronotum (Fig. 23): Slightly transverse (PWmax/PL = 1.45), distinctly narrowed basad (PWmax/PWmin =1.24) and widest in anterior third. Sides rounded in anterior two-thirds and slightly sinuate before base; each side with one lateral setigerous pore in anterior third. Apical margin slightly emarginate, bordered only laterally. Basal margin almost straight, distinctly bordered throughout, approximately equal in width to apical margin and slightly narrower than elytral base between humeral angles. Apical angles slightly protruded anteriad, acute and very narrowly rounded at apices. Basal angles obtuse, with sharp, slightly denticulate apices. Pronotal disc moderately convex, not depressed basally, markedly sloped toward apical angles in apical half and slightly sloped toward sides in basal half. Lateral depressions very narrow from apical angles to pronotal base. Basal foveae oval and shallow, isolated from basal border. Areas at basal angles and between basal foveae slightly convex. Pronotal surface punctate almost throughout, very finely punctate medially and along apical margin and more densely and coarsely punctate basally and along sides, punctures confluent and particularly coarse within basal foveae.

Elytra: Oval, markedly rounded at sides, comparatively short and wide (EL/EW= 1.34, EL/PL = 2.38, EW/PWmax = 1.22), widest at middle, not fused along suture. Humeri not prominent, with a tiny acute denticle at apices (visible only from behind). Subapical sinuations distinct, moderately deep. Sutural angle slightly less than 90°, slightly blunted at apex. Basal borders slightly sinuate to humeri, joined with lateral margin at an obtuse angle. Striae impunctate, thin, superficial along their entire length. Parascutellar strioles present but short, basal setigerous pore present. Intervals flat up to apices, impunctate. Umbilicate setal series divided into two groups (humeral and apical), with an isolated setigerous pore medially.

Hindwings: Reduced to small scales.

Venter: Prosternum glabrous. Medial prosternal process slightly projected posteriad (Fig. 25). Proepisterna smooth. Metepisterna (Fig. 24) approximately as long as wide, markedly narrowed posteriad. Sternum VII (anal) with two pairs of setae along apical margin, widely rounded at apex.

Legs: Metacoxae (Fig. 26) without posteromedial setigerous pore or any additional setigerous or nonsetigerous foveae medially. Metafemora with two setae along posterior margin. Protibiae with 1 stouter and 1–2 slenderer spines at outer distal margin and with one ventroapical spine. Tarsi glabrous dorsally, tarsomere 5 with three or four pairs of lateroventral setae. Metatarsi short, shorter than width of head just behind eyes, with tarsomere 1 about 1.4 times as long as tarsomere 2 and notably shorter than tarsomeres 2+3. Male protarsi markedly enlarged (tarsomeres 2 and 3 much wider than long and tarsomeres 1–4 with adhesive vestiture ventrally); mesotarsi moderately enlarged (tarsomere 1 slightly longer than wide and lacking adhesive vestiture ventrally; tarsomere 2 approximately as long as wide and with adhesive vestiture ventrally; tarsomere 3 about 1.2 times as wide as long and with adhesive vestiture ventrally; and tarsomere 4 distinctly smaller than tarsomeres 2 and 3, deeply concave apically and without adhesive vestiture ventrally).

Aedeagus (Figs 27–29): Median lobe asymmetrical, markedly bent ventrad behind basal bulb, more faintly bent ventrad in apical portion, and slightly directed dorsad just at apex to form a faintly recognizable apical capitulum; middle portion of ventral margin convex. Terminal lamella (Fig. 27) about 2.0 times as long as wide, flat, triangular, slightly blunted at apex (dorsal aspect) and distinctly directed to right, its dorsal side with a triangular depression in basal quarter. Apical orifice in dorsal position, prolonged to basal bulb. Internal sac with two small and narrow spiny patches basally.

#### Distribution.

Fig. 56. Known only from the type locality (Hanmi) in the eastern Himalaya, in the southeastern part of Xizang Autonomous region (Tibet), Medog County, China.

#### Specific epithet.

 The specific epithet refers to Hanmi, the locality in Tibet, from where the new species is described.

#### Remarks.

 This new species belongs to the *semenowi* group (sensu Kataev and Schmidt 2006), members of which are characterized by metacoxae without additional posteromedial setigerous pores and the aedeagus with a flat, triangular terminal lamella. Within this group, *Chydaeus hanmiensis* adults are distinguished by the slightly sinuate pronotal sides combined with comparatively short metepisterna (approximately as long as wide). In addition, the prosternal medial process is slightly projected posteriad in *Chydaeus hanmiensis* members, whereas this process is not or only faintly projected in members of other species of the *semenowi* group. The holotype of *Chydaeus hanmiensis* is externally similar to males of *Chydaeus obtusicollis* Schauberger, 1932, but, in the latter, the medial lobe is more arcuate and with an additional medial spiny patch in the internal sac on the left side.

### Chydaeus asetosus

Kataev & Kavanaugh sp. n.

urn:lsid:zoobank.org:act:E568BCDF-110A-4CDD-A644-0F4C7A0507F9

http://species-id.net/wiki/Chydaeus_asetosus

Figs 30–37 54 57 67

#### Type material.

 Holotype, a male, deposited in IOZ, labeled: “China, W Yunnan, NE Fugong, 26°57'09"N, 98°54'00"E – 26°56'30"N, 98°55'14"E, 2160–2690 m, 29.V.2006, I. Belousov & I. Kabak leg.". A total of 22 paratypes (16 males and 6 females, including 5 males and 2 females in CAS, 5 males and 2 females in IOZ, and 6 males and 2 females in ZIN and cBL&KB) from the following localities: **China. Yunnan Province**. *Fugong County:* 3 males, 2 females , same data as holotype, 29.V.2006, I. Belousov & I. Kabak leg. (ZIN, cBL&KB); 3 males, W Yunnan, NE Fugong, 26°56'51"N, 98°54'12"E, 2190 m, 29.V.2006, I. Belousov & I. Kabak leg. (ZIN, cBL&KB); 1 male, 2 females, Fugong, Lishadi, 0.3 km above Shibali on Yaping road, 27.16337°N, 98.78208°E, 2475 m, 7.V.2004, D. Kavanaugh leg. (IOZ, CAS); 1 male [teneral], Fugong, Lishadi Town, 4 km below Shibali, road, 27.15727°N, 98.79784°E, 2280 m, 11.VIII.2005, H.B. Liang leg. (IOZ); 1 male, Fugong, Lishadi, Shibali, around hotel, 27.16530°N, 98.77980°E, 2530 m, 4.VIII.2005, H.B. Liang leg. (IOZ); 1 male [teneral], Fugong, Lishadi Town, 0.5 km below Shibali, 27.16520°N, 98.77980°E, 2530 m, 5.VIII.2005, H.B. Liang & G. Tang leg. (CAS); 1 male, Fugong Co., Lumadeng, 5 km below Shibali, road, 27.16520°N, 98.77980°E, 2190 m, 7.V.2004, X.-Y. Li & M. Xie leg. (IOZ); 1 male, Fugong Co., Lumadeng, Yaping - Shibali, roadside, 27.14627°N, 98.81559°E, 2030 m, 3.V.2004, H.B. Liang leg. (CAS); 1 male, Fugong Co., Lumadeng, Yaping - Shibali, 11 km, 27.13839°N, 98.82147°E, 1850 m, 25.IV.2004, H.B. Liang & M. Xie leg. (CAS); 3 males, 2 females, Fugong Co., Lumadeng, Yaping, Yejiadi, roadside, 27.08004°N, 98.77325°E, 2307 m, 10.V.2004, H.B. Liang & B.-X. Zhu leg. (IOZ, CAS).

#### Description.

 Dorsal habitus as in Fig. 54. Size: Body length 8.8-10.8 mm, width 3.8-4.6 mm.

Color: Body black, shiny on dorsum; labrum, also mandibles basally and lateral bead of pronotum in many specimens paler, reddish brown; antennae, palpi, tibiae, and tarsi reddish brown, femora blackish brown.

Microsculpture: Head with dorsal microsculpture present throughout in most specimens, comprised of fine isodiametric meshes, more or less effaced on frons and vertex. Pronotum with microsculpture comprised of more or less effaced meshes, more distinct in females than in males. Elytral microsculpture in males comprised of more or less isodiametric meshes, distinct on two or three lateral intervals and in area along basal border, otherwise more or less effaced; in females comprised of isodiametric, nearly granulate meshes in basal half and on two or three lateral intervals apically and of slightly transverse meshes on inner intervals in apical half.

Head: Comparatively large (HWmax/PWmax = 0.70–0.73 and HWmin/PWmax = 0.60-0.66), with micropunctures in areas near frontal foveae and around and behind supraorbital setae in most specimens, micropunctures also present on clypeus and frons in some specimens, micropunctation absent from head in a few specimens; tempora short, nearly flat, sloped to neck. Clypeus slightly concave and distinctly bordered apically. Frontal suture distinct, superficial or slightly impressed. Frontal foveae small and shallow, clypeo-ocular prolongations superficial, short in most specimens but distinct to supraorbital furrows in some specimens. Supraorbital seta situated at level of hind margin of each eye. Eyes small, moderately convex. Antennae short, not extended to pronotal basal margin, with antennomeres 5 to 7 each about 1.6–2.0 times as long as wide. Labrum distinctly emarginate apically. Left mandible truncate at apex. Ligular sclerite slightly widened and rounded at apex.

**Figures 30–37.**
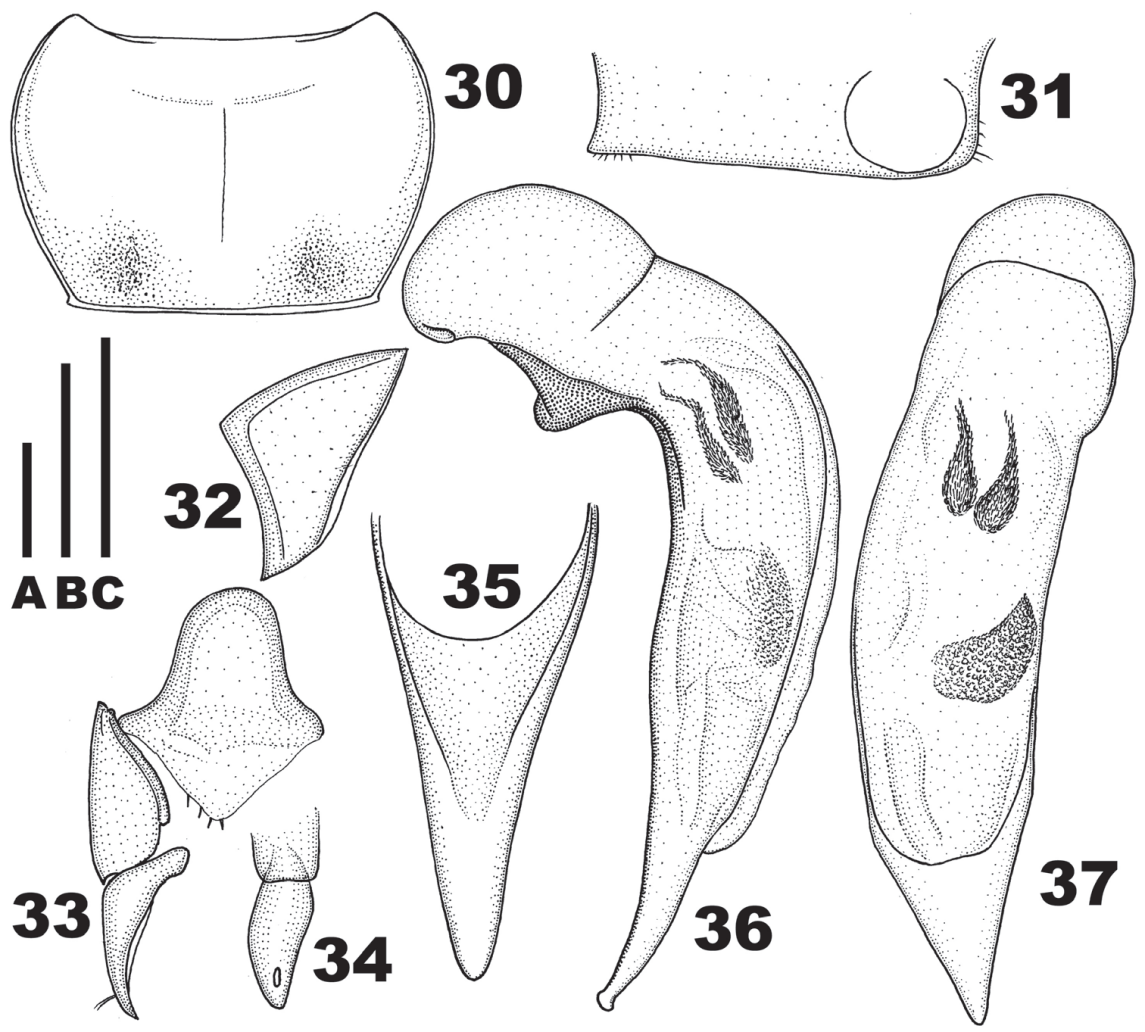
*Chydaeus asetosus* sp. n. (holotype) **30** Pronotum **31** Ventral side of prosternum, right lateral view **32** Left metepisternum **33** Right hemisternite and stylus, ventral view **34** Stylus, lateral view **35** Terminal lamella of median lobe, dorsal view **36** Median lobe, left lateral view **37** Median lobe, dorsal view. Scale lines: **A** = 1.0 mm (Fig. 30), **B** = 0.5 mm (Fig. 35), **C** = 1.0 mm (Figs 31–34, 36–37).

Pronotum (Fig. 30): Slightly transverse (PWmax/PL = 1.40–1.51), narrowed basad (PWmax/PWmin =1.25–1.36) and widest in anterior third. Sides rounded along their entire length, but in basal half less distinctly than in apical half; without any lateral setigerous pores. Apical margin very slightly concave or nearly straight medially, bordered only laterally. Basal margin more or less straight (very broadly rounded in some specimens), distinctly bordered throughout, width approximately equal to apical margin and slightly narrower than elytral base between humeral angles. Apical angles nearly 90° (lateral aspect), slightly protruded anteriad. Basal angles obtuse, each with small denticle at apex. Pronotal disc convex, only faintly depressed basally, markedly sloped to apical angles. Lateral depressions varied, from very narrow along entire length, indistinct, and with area at basal angles convex, to distinctly widened and deepened in basal third and depressed at basal angles. Basal foveae small, either flat or slightly impressed. Pronotal surface densely and distinctly punctate, mainly along sides laterobasally and lateroapically, with punctation more widely distributed over entire basal and apical portions in some specimens, or with very fine punctures also present in central portion in a few specimens, or with punctation restricted only to lateral and latero-basal areas in a few other specimens; in all specimens, punctures coarsest in latero-basal portion and in narrow area along sides.

Elytra: Oval, rounded at sides, moderately wide (EL/EW= 1.33–1.44, EL/PL = 2.42–2.67, EW/PWmax = 1.22–1.30), widest at middle, not fused along suture. Humeri subangulate, rounded at apices, each with a tiny denticle visible only from behind. Subapical sinuations moderately deep. Sutural angles not separated from each other medially, slightly less than 90°, with apices blunted in male and sharp in female. Basal borders slightly sinuate, joined with lateral margin at very obtuse angle. Striae impunctate, slightly impressed along entire length. Parascutellar striae present, short, basal setigerous pores present; in some specimens, first (sutural) striae interrupted basally with distal part of parascutellar striae connected to proximal part of isolated distal portion of first striae. Intervals slightly convex or nearly flat, in some specimens two or three lateral intervals very finely micro-punctate. Umbilicate setal series more or less widely interrupted at middle.

Hindwings: Reduced to small scales.

Venter: Prosternum smooth and glabrous, with at most a few very fine and barely evident setae apically. Prosternal medial process slightly prominent, not projected posteriad (Fig. 31). Proepisterna smooth, at most finely micropunctate. Metepisterna (Fig. 32) markedly narrowed posteriad, approximately as long as wide or slightly wider than long. Sternum VII (anal) in both sexes with two pairs of setae along apical margin and rounded at apex. Tergum VII (anal) of female rounded at apex.

Legs: Metacoxae generally without posteromedial setigerous pore or any additional setigerous or nonsetigerous foveae medially [in one female collected at 0.3 km above Shibali on Yaping road, left metacoxa with an additional posteromedial setigerous pore]. Metafemora with two setae along posterior margin. Protibiae with one ventroapical spine, outer margin with one or two stouter spines and also two or three slenderer spines apically in most males, with four or five uniform, stout spines in females. Tarsi glabrous dorsally, tarsomere 5 with three (four in some specimens) pairs of lateroventral setae. Metatarsi approximately equal in length to minimum linear distance across neck constriction just behind eyes in males, slightly shorter in females; tarsomere 1 distinctly longer than tarsomere 2, but distinctly shorter than tarsomeres 2+3. In males, protarsi markedly enlarged (tarsomeres 2-4 much wider than long, tarsomere 1 about as long as wide, and tarsomeres 1-4 with adhesive vestiture ventrally); mesotarsi moderately enlarged (tarsomere 1 slightly longer than wide; tarsomere 2 approximately as long as wide; tarsomere 3 about 1.3 times as wide as long; and tarsomere 4 distinctly smaller than tarsomeres 2 and 3, and deeply concave apically, and tarsomeres 2–4 with adhesive vestiture ventrally).

Female genitalia (Figs 33–34): Apical stylomere comparatively faintly curved.

Aedeagus (Figs 35–37): Median lobe markedly bent ventrad behind basal bulb and convex on ventral side medially. Terminal lamella directed ventrad, triangular in dorsal aspect, about two times as long as wide and very narrowly rounded at apex (Fig. 35), dorsal side basally with large triangular depression prolonged apicad up to or beyond middle of terminal lamella; apical capitulum very small, slightly prominent ventrad and dorsad. Apical orifice slightly shifted to right, prolonged to basal bulb. Internal sac with two basal and (in most specimens) one medial spiny patches; medial spiny patch, if present, located on right side of medial lobe.

#### Geographical variation.

 Specimens examined from the area around Shibali are smaller on average (body length 8.8–10.2 mm, mean = 9.7 mm) than the specimens from northwest of Fugong (body length 9.7–10.8 mm, mean = 10.4 mm) and their pronota are slightly narrower [PWmax/PL = 1.40–1.43 (mean = 1.41) and 1.42–1.50 (mean = 1.48), respectively] and with deeper basal foveae.

#### Distribution.

Fig. 57. This new species is known only from the northern part of the Gaoligong Shan (Fugong County) in northwestern Yunnan Province, China.

#### Habitat.

 Specimens were collected in roadside and road cut open areas (Fig. 67) and in other disturbed areas, hidden under stones and other debris during daylight hours and active on the soil surface at night.

#### Specific epithet.

 The specific epithet refers to the asetose sides of the pronotum in members of this species.

#### Remarks.

 Like members of *Chydaeus hamiensis*, those of *Chydaeus asetosus* have the metacoxae without additional posteromedial setigerous pores and the aedeagus with flat, triangular terminal lamella, which suggest that they belong to the *semenowi* group (sensu Kataev and Schmidt 2006). Members of this new species are easily distinguished from those of all known species of *Chydaeus* (not just of the *semenowi* group species) by the absence of lateral pronotal setae. Members of all other *Chydaeus* species known to us have from one to several lateral setae on each pronotal side. Among the species of the *semenowi* group, *Chydaeus asetosus* members are similar in habitus and male genitalia to members of *Chydaeus obtusicollis*, but the median lobe is less markedly arcuate, the metepisterna are much shorter, the body is more convex, and the pronotum is relatively larger in *Chydaeus asetosus* members than in *Chydaeus obtusicollis* members. The short metepisterna and male genitalia of *Chydaeus asetosus* members are similar to those of *Chydaeus hanmiensis* members, but the former differ from the latter in having rounded pronotal sides, impressed elytral striae, and a longer terminal lamella of the aedeagus. In external morphology, *Chydaeus asetosus* adults are also similar to those of *Chydaeus satoi* Ito, 2003; but in males of latter species, the median lobe has the terminal lamella much longer and more markedly bent dorsad.

### Chydaeus baoshanensis

Kataev & Liang sp. n.

urn:lsid:zoobank.org:act:EDE8E8EE-75B2-48E7-9B56-ED540BA92033

http://species-id.net/wiki/Chydaeus_baoshanensis

Figs 38–43 55 57

#### Type material.

 Holotype, a male, in ZIN, labeled: “**China**, Yunnan, N Baoshan, 25°30'10"N, 99°06'40"E – 25°29'26"N, 99°06'16"E, 2265–2530 m, 08.V.2006, I. Belousov & I. Kabak leg.” .

#### Description

 (male). Dorsal habitus as in Fig. 55. Size: Body length 9.5 mm, width 4.1 mm.

Morphological characters as described for *Chydaeus asetosus* except as follows:

Color: Body black, shiny on dorsum; labrum, tibiae and tarsi paler, blackish brown; antennae dark brown, palpi lighter brown.

Microsculpture: Head with dorsal microsculpture effaced. Pronotum with microsculpture more or less effaced.

Head: Large (HWmax/PWmax = 0.71 and HWmin/PWmax = 0.62), covered with very fine micro-punctures on labrum and on areas around frontal foveae, above supraorbital furrows, and around and behind supraorbital setae. Frontal suture thin, slightly impressed. Frontal foveae very shallow, clypeo-ocular prolongations extended to supraorbital furrows. Ligular sclerite with apical angles slightly projected laterally.

**Figures 38–43.**
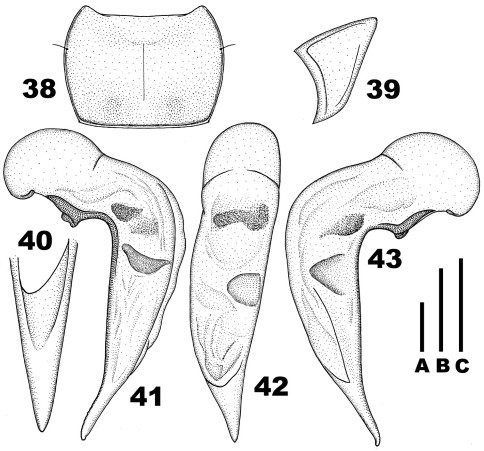
*Chydaeus baoshanensis* sp. n. (holotype) **38** Pronotum. **39** Left metepisternum **40** Terminal lamella of median lobe, dorsal view **41** Median lobe, left lateral view **42** Median lobe, dorsal view **43** Median lobe, right lateral view. Scale lines: **A** = 1.0 mm (Fig. 38), **B** = 0.5 mm (Fig. 40), **C** = 1.0 mm (Figs 39, 41–43).

Pronotum (Fig. 38): Slightly transverse (PWmax/PL = 1.47), slightly narrowed basad (PWmax/PWmin =1.23), with one lateral seta on each side before middle. Basal margin nearly straight medially and slightly oblique laterally, slightly wider than apical margin. Lateral depressions very narrow and barely evident along entire length. Basal foveae absent and area near basal angles slightly convex, almost flat. Pronotal surface punctate almost throughout, but punctures extremely fine medially.

Elytra: Elytra relatively short (EL/EW= 1.40, EL/PL = 2.45, EW/PWmax = 1.19), widest just behind middle, without humeral denticles. Subapical sinuations slightly shallower than in *Chydaeus asetosus*. Parascutellar striae short, about 2.0-3.5 times as long as diameter of parascutellar setigerous pore, not connected distally with first (sutural) striae. Intervals nearly flat, lateral intervals very finely and indistinctly micro-punctate. Umbilicate setal series without distinct gap at middle.

Venter: Metepisterna (Fig. 39) markedly narrowed posteriad, slightly wider than long.

Legs: Metatarsi slightly shorter than minimum linear distance across neck constriction just behind eyes

Aedeagus (Figs 40-43): Median lobe similar to that of *Chydaeus asetosus*, but terminal lamella (Fig. 40) narrower, slightly curved dorsad at apex and without apical capitulum; basal triangular depression on its dorsal side prolonged apicad up to middle of terminal lamella. Internal sac with two basal and one medial spiny patches; medial spiny patch located on left side of median lobe .

#### Distribution.

Fig. 57. This new species is known only from the type locality, N Baoshan (Baoshan City) in the southern part of the Nu Shan (Mountains) in western Yunnan Province, China.

#### Specific epithet.

 The specific epithet refers to Baoshan city in Yunnan Province, where the type specimen was collected.

#### Remarks.

*Chydaeus baoshanensis* belongs to the *semenowi* group and the holotype specimen is morphologically very similar to specimens of *Chydaeus asetosus*, differing from them mainly in the presence of one lateral seta on each pronotal side, the wider pronotal base and form of the male genitalia. The terminal lamella of the aedeagus of *Chydaeus baoshanensis* males is narrower than that of *Chydaeus asetosus*males, slightly curved dorsad and without an apical capitulum. As with *Chydaeus asetosus*, *Chydaeus baoshanensis* members are very similar externally to those of *Chydaeus satoi* and *Chydaeus hanmiensis*, including in having short metepisterna; but males of all four species are very distinct in genitalic form. In addition, *Chydaeus baoshanensis* differs from *Chydaeus hanmiensis* in having deeper elytral striae, rounded, not sinuate, pronotal sides, and the prosternal medial process not projected posteriad. Members of *Chydaeus baoshanensis* differ most distinctly from those of *Chydaeus obtusicollis* in having much shorter metepisterna.

### Chydaeus satoi

Ito, 2003

http://species-id.net/wiki/Chydaeus_satoi

Figs 44–46 58 64

#### Material examined.

 A total of 14 specimens (9 males and 5 females, including 4 males and 2 females in CAS, 4 males and 2 females in IOZ; and 1 male and 1 female in ZIN) were examined from the following localities: **China. Yunnan Province**. *Lushui County:* 5 males, 2 females, Luzhang, Yaojiaping, riverside, 25.97722°N, 98.71091°E, 2527 m, 20.V.2005, D. Kavanaugh & D.Z. Dong leg. (CAS, IOZ); 1 male, same data, but 19.V.2005 (IOZ); 3 males, 3 females, Luzhang, Yaojiaping, roadside, 25.97526°N, 98.71000°E, 2515 m, 20.V.2005, H.B. Liang leg. (CAS, IOZ, ZIN).

**Figures 44–46.**
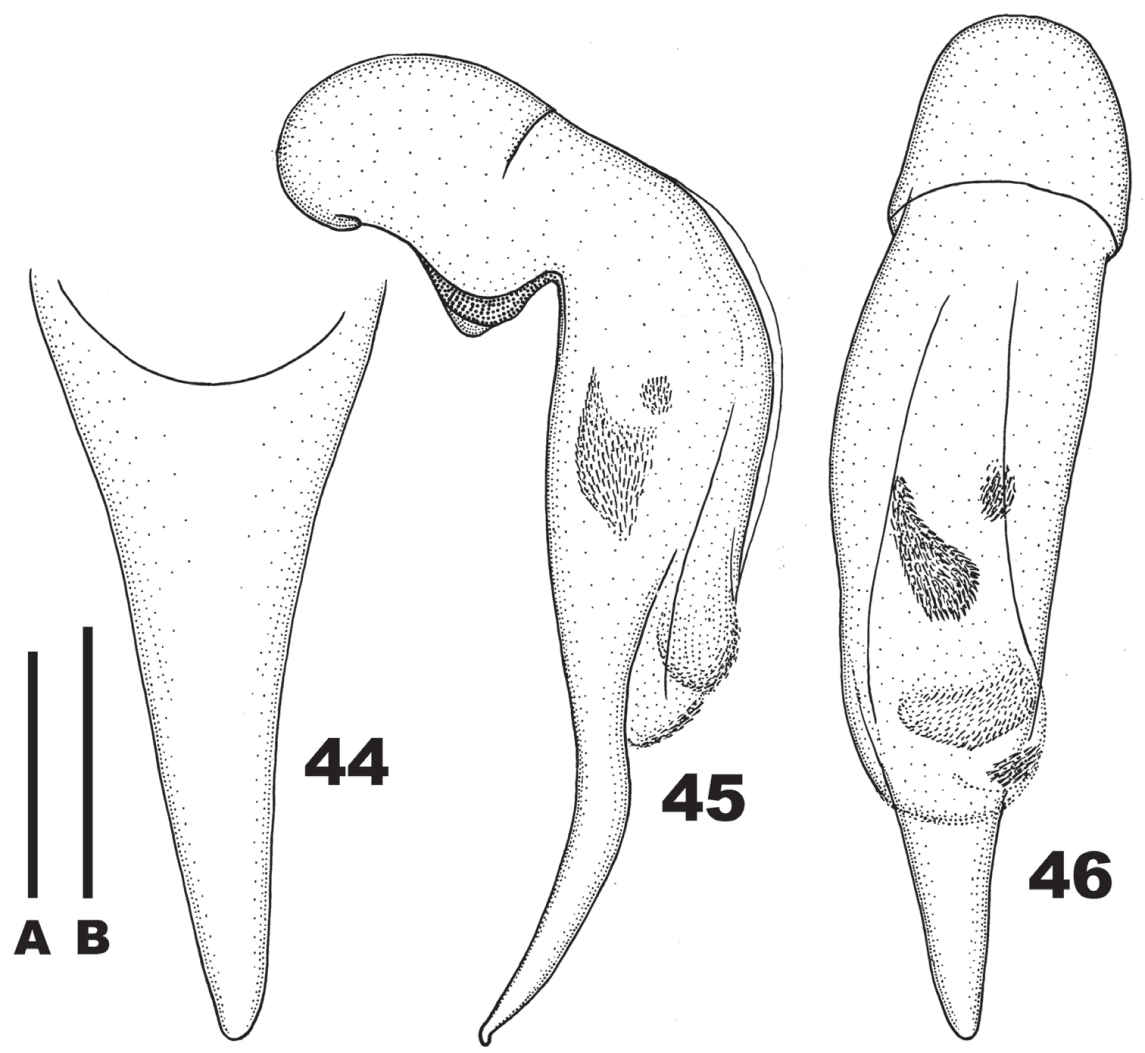
*Chydaeus satoi* Ito (Luzhang, Yaojiaping) **44** Terminal lamella of median lobe, dorsal view **45** Median lobe, left lateral view **46** Median lobe, dorsal view. Scale lines: **A** = 0.5 mm (Fig. 44), **B** = 1.0 mm (Figs 45–46).

#### Distribution.

Fig. 58. *Chydaeus satoi* was previously known only from the type locality (Zhonghe Feng) at an elevation of 2620 m in the Diangcang Shan (Mountains), situated north of Dali, western Yunnan Province, China (Ito, 2003). The additional material listed above was also collected in western Yunnan, but in the Gaoligong Shan, about 150 km to the west of the type locality.

#### Habitat.

 Specimens were collected in roadside and road cut open areas (Fig. 64) and on open, disturbed stream banks, hidden under stones and other debris during daylight hours.

#### Remarks.

 This species belongs to the *semenowi* group (Kataev and Schmidt 2006). Male members are very similar in their appearance to those of *Chydaeus baoshanensis*, but easily distinguished from them by the peculiar median lobe with the terminal lamella very long and markedly bent ventrad (Figs 44–46).

In male specimens from the Gaoligong Shan, the terminal lamella is slightly narrower in dorsal aspect than in males from the Diangcang Shan as illustrated by Ito (2003). The significance of this difference should be tested by examination of additional material. One of us (HBL) examined the type specimens of *Chydaeus satoi* at Ito’s home (Kawanishi City, Japan) and found no differences between the type and specimens from the Gaoligong Shan.

### Chydaeus obtusicollis

Schauberger, 1932

http://species-id.net/wiki/Chydaeus_obtusicollis

Figs 57 68

#### Material examined.

 A total of 95 specimens (49 males and 46 females, including 23 males and 23 females in CAS, 24 males and 22 females in IOZ, and 2 males and one female in ZIN) were examined from the following localities: **China**. **Xizang Autonomous Region**: *Cona County*: 1 female, 2000 m, 7.VIII.1974 (IOZ). *Medog County*: 1 male, Medog, Baibung, 850 m, 17.III.1983 (IOZ). **Yunnan Province**. *Gongshan County*: 1 female, Dulongjiang, Bapo, along roadside, 27.73902°N, 98.34975°E, 1412 m, 3.XI.2004, H.B. Liang leg. (IOZ); 2 males, 2 females, Gongshan, Dulongjiang, 1 km S of Bapo, 27.73453°N, 98.35042°E, 1333 m, 3.XI.2004, V.F. Lee leg. (IOZ, CAS); 1 male, Dulongjiang, Bapo, along roadside, 27.73902°N, 98.34975°E, 1412 m, 26.X.2004, H.B. Liang leg. (IOZ); 1 female, Dulongjiang, Bapo, Mabiluo, riverside, 27.76208°N, 98.34567°E, 1503 m, 27.X.2004, H.B. Liang leg. (IOZ); 3 males, 2 females, Dulongjiang, Maku village, roadside, 27.68533°N, 98.30425°E, 1823 m, 2.XI.2004, H.B. Liang leg. (CAS, IOZ, ZIN); 1 male, same data, but 1.XI.2004 (IOZ); 2 females, Dulongjiang, Maku village, roadside, 27.68533°N, 98.30425°E, 1823 m, 1.XI.2004, H.B. Liang leg.(CAS, IOZ); 1 female, Dulongjian, 0.5 km WSW Maku, trail, 27.68310°N, 98.30038°E, 1845 m, 29.VIII.2006, D. Kavanaugh leg. (CAS); 1 male, Dulongjiang, Lawaduo, roadside, 27.69666°N, 98.34934°E, 1466 m, 4.XI.2004, H.B. Liang leg. (IOZ); 2 females, Dulongjiang, Kongdang, roadside, 27.87696°N, 98.33587°E, 1525 m, 5.XI.2004, H.B. Liang leg. (IOZ); 3 males, 3 females, Dulongjiang, Kongdang, roadside, 27.87696°N, 98.33587°E, 1525 m, 25.X.2004. H.B. Liang leg. (CAS, IOZ); 8 males, 3 females, Cikai Town, 0.5 km of Kongdang, 27.88111°N, 98.34063°E, 1500 m, 25.X.2004, D. Kavanaugh leg. (CAS, IOZ); 1 male, Dulongjiang, Bapo, along roadside, 27.73902°N, 98.34975°E, 1412 m, 26.X.2004, H.B. Liang leg. (IOZ); 3 males, Dulongjiang, Pengjiwang, above Bapo, 27.72999°N, 98.40650°E, 2250 m, 28.X.2004, H.B. Liang leg. (CAS, IOZ, ZIN); 1 male, Dulongjiang, Miliwang, above Bapo, 27.72383°N, 98.36117°E, 1956 m, 31.X.2004, H.B. Liang leg. (IOZ); 3 males, 2 females, Dulongjiang, 0.6 km N Dizhengdang, 28.08442°N, 98.32652°E, 1880 m, 29.X.2004, D. Kavanaugh leg. (CAS, IOZ); 1 female, Dulongjiang, S edge of Dizhengdang, 28.07654°N, 98.32603°E, 1890 m, 29.X.2004, D. Kavanaugh leg. (ZIN); 1 male, same data, but 28.X.2004 (IOZ); 1 male, Dulongjiang, Dizhengdang, Silalong He, 28.07654°N, 98.32603°E, 1890 m, 30.X.2004, D. Kavanaugh & D.Z. Dong leg. (CAS); 1 male, 1 female, Dulongjiang, 2.8 km S Longyuan Vill., 28.00905°N, 98.32204°E, 1660 m, 31.X.2004, D. Kavanaugh & D.Z. Dong leg. (CAS); 3 males, 3 females, Dulongjiang, 2.3 km S Longyuan, 28.00532°N, 98.32145°E, 1685 m, 2.XI.2004, D. Kavanaugh leg. (CAS, IOZ); 5 males, 8 females, Dulongjiang, Elidang Village, beach, 28.00287°N, 98.32145°E, 1640 m, 3.XI.2004, D. Kavanaugh & D.Z. Dong leg. (CAS, IOZ); 3 males, 4 females, Dulongjiang, Xianjiudang Village, 27.94092°N, 98.33340°E, 1580 m, 4.XI.2004, D. Kavanaugh & D.Z. Dong leg. (CAS, IOZ); 1 male, Dolongiang Township, Moqie Wang at Gongshan-Dulong Road km 91, 1550 m, 27.90085°N, 98.34721°E, 6.XI.2004, Stop DHK-2004-077, D. Kavanaugh & H.B. Liang leg. (IOZ); 1 male, Dulongjiang, 0.2 km S Mukewang He, 27.84125°N, 98.32979°E, 1450 m, 7.XI.2004, D. Kavanaugh & D.Z. Dong leg. (CAS); 5 males, 8 female, Dulongjiang, 0.5 km N Kongdang, 27.88111°N, 98.34062°E, 1500 m, 7.XI.2004, D. Kavanaugh leg. (CAS, IOZ); 2 females, Dulongjiang, Moqiewang He, 27.91040°N, 98.41076°E, 2185 m, 8.XI.2004, D. Kavanaugh & M. Dixon leg. (IOZ). *Lushui County:* 1 male, Nujiang Prefecture, Gangfang sancha lukou, 26.073°N, 98.345°E, 1500 m, 14–15.X.1998, D.H. Kavanaugh, C.E. Griswold, C. Ferraris & C.L. Long leg. (IOZ).

#### Distribution.

Fig. 57. All the previous records for species were restricted to the Central Himalaya, from the eastern part of Nepal to West Bengal (Darjeeling) and Sikkim, at elevations of 1800–2900 m (Kataev and Schmidt 2006). Specimens recorded here extend the range of *Chydaeus obtusicollis* eastward to Xizang Autonomous Region (Cona and Medog counties) and northwestern Yunnan Province (Gongshan and Lushui counties), China. The elevational range of Yunnan records extends from 1400 to 2250 m.

#### Habitat

**.** Specimens were collected in roadside and road cut open areas (Fig. 68), on open, disturbed stream banks, and in other disturbed areas, hidden under stones and other debris during daylight hours and active on the soil surface at night.

#### Remarks.

Kataev and Schmidt (2006) included *Chydaeus obtusicollis* in the *semenowi* group and redescribed this species based on Central Himalayan specimens. Specimens examined from China are similar to those from the Central Himalaya in the main morphological characteristics, including the male genitalia; but the basal pronotal angles, in some specimens from Yunnan, have more or less distinct apical denticles. The taxonomic status of specimens from Yunnan merits further study. As with populations in the Central Himalaya, Yunnan populations of *Chydaeus obtusicollis* are dimorphic for wing length, with both brachypterous and macropterous individuals present.

### Chydaeus convexus

Ito, 2002

http://species-id.net/wiki/Chydaeus_convexus

Figs 58 70

#### Material examined.

 A total of 125 specimens (71 males and 54 females, including 35 males and 26 females in CAS, 35 males and 27 females in IOZ, and 1 male and 1 female in ZIN) were examined from the following localities: **China**. **Yunnan Province**. *Longling County*: 1 female, Longjiang, Xiaoheishan, tree & log, 24.83696°N, 98.75735°E, 2120 m, 27.V.2005, H.B. Liang & J.L Yang leg. (IOZ); 1 male, same data, but riverside, 24.82886°N, 98.75917°E, 2010 m, 25.V.2005, H.B. Liang leg. (IOZ); 3 males, 1 female, same data, but roadside, 24.82888°N, 98.76001°E, 2020 m, 28.V.2005, D. Kavanaugh & D.Z. Dong leg. (CAS); 2 female, same data, but riverside, 26.V.2005, D. Kavanaugh leg. (CAS). *Longyang County (District)*: 1 female, Bawan, 34 km from Bawan on Tengchong Road, 2310 m, 24.92944°N, 98.75917°E, 16.X.2003, D.Z. Dong leg. (IOZ); 1 male, Bawan, Luokeng, 41 km on road to Tengchong, 24°56'23.2"N, 98°45'11.6"E, 2440 m, 15.X.2003, day, H.-B. Liang & X.C. Shi leg. (IOZ); 1 female, Bawan, 41 km on old road to Tengchong, 25°56'15.0"N, 98°45'02.8"E, 2486 m, 11.X.2003, H.B. Liang leg. (IOZ); 1 male, Gaoligong Shan, Baoshan Pref., Nankang Yakou, 24°49.9'N, 98°46.0'E, 2130 m, 4-7 IX.1998, Stop 98–129A, D. Kavanaugh, C.E. Griswold, C.L. Long, R. Li & H.-X. He leg. (CAS); 1 male, same data as preceding (IOZ); 1 female, same data, but 24.81944°N, 98.77111°E, 2130 m, 27.X.2003, D.Z. Dong leg. (IOZ); 1 male,1 female, Bawan Town, Nankang Yakou, roadside, 24°49'33.4"N, 98°46'20.0"E, 2130 m, 26.X.2003, H.B. Liang & X.C. Shi leg. (IOZ); 1 male, 6 females, same data, but 24.81944°N, 98.77111°E, 2130 m, 31.X.2003, D.Z. Dong leg. (CAS, IOZ); 5 males, 4 females, Bawan Town, Nankang forest station, 24°49'28.8"N, 98°46'43.6"E, 2085 m, 27.X.2003, H.B. Liang & X.C. Shi leg. (CAS, IOZ); 1 female, Bawan, Nankang station, 24.82614°N, 98.77602°E, 1900 m, 26.V.2005, D.Z. Dong leg. (IOZ); 1 female, same data, but 24.83178°N, 98.76462°E, 2180 m, 22.V.2005, D. Kavanaugh & D.Z. Dong leg. (CAS); 6 males, 4 females, Bawan, 36-37 km on old road to Tengchong, 24°56'03.3"N, 98°46'46.4"E, 2150 m, 12.X.2003, H.B. Liang & X.C. Shi leg. CAS, IOZ); 1 male, 3 females, Bawan, Bawan - Tengchong Road km 29-35, 2000-2350 m, 24.92916°N, 98.75861°E, 12.X.2003, D.Z. Dong leg. (CAS, IOZ); 1 male, 4 females, Bawan, Yakou to Sanchawa, along road, 24°56'50.5"N, 98°45'20.0"E, 2300 m, 13.X.2003, H.B. Liang & X.C. Li leg. (CAS, IOZ); 4 males, Bawan, Dasheyao forest station - Yakou, 24°55'37.4"N, 98°45'09.8"E, 2404 m, 12.X.2003, H.B. Liang & X.C. Shi leg. (CAS, IOZ); 1 male, 2 females, Bawan, Dasheyao, 24.92989°N, 98.75862°E, 2320 m, 1.VI.2005, Dao Zhilong leg. (CAS, IOZ); 2 males, same data, but 24.92994°N, 98.75850°E, 2300 m, 3.VI.2005, D. Kavanaugh & D.Z. Dong leg. (CAS, IOZ); 2 males, 2 females, Bawan, Nankang station, 24.82260°N, 98.78201°E, 2060 m, 23.V.2005, H.B. Liang leg. (CAS, IOZ); 1 male, same data, but 24.82600°N, 98.77690°E, 2090 m, 28.V.2005, D. Kavanaugh leg. (CAS); 1 female, same data, but 24.82587°N, 98.76832°E, 2048 m, 22.V.2005, H.B. Liang leg. (IOZ); 1 female, same data, but 24.83178°N, 98.76462°E, 2180 m, 22.V.2005, D. Kavanaugh & D.Z. Dong leg. (CAS); 1 female, same data, but 24.82614°N, 98.77602°E, 1900 m, 26.V.2005, D.Z. Dong leg. (IOZ); 2 males, Bawan, Luoshuidong, 2300-2480 m, 24.93278°N, 98.75333°E, 13.X.2003, D.Z. Dong leg. (CAS, IOZ); 11 males, 4 females, Bawan, Sanchahe, 24.94755°N, 98.75564°E, 2300 m, 3.VI.2005, H.B. Liang & H.M. Yan leg. (CAS, IOZ); 4 males, same data, but 24.94865°N, 98.75193°E, 2350 m, 30.V.2005, H.B. Liang leg. (CAS, IOZ); 1 male, same data, but 2300 m, 24.94849°N, 98.75699°E, 30.V.2005, D. Kavanaugh leg. (IOZ); 2 males, 2 females, same data, but 3.VI.2003, D. Kavanaugh & D.Z. Dong leg. (CAS, IOZ); 1 female, same data, but D.Z. Dong leg. (IOZ). *Tengchong County*: 1 male, Jietou, Datang, Dahetou, 25.69700°N, 98.68059°E, 1800-2000 m, 16.VI.2005, Huang Hao leg. (IOZ); 8 males, 6 females, Dahaoping, 46-51 km on old road, 24°57'25.6"N, 98°44'12.3"E, 2220 m, 17.X.2003, H.B. Liang & X.C. Shi leg. (IOZ, ZIN); 2 males, Dahaoping, 43 km from Bawan on Tengchong Road, 2410 m, 24.95361°N, 98.73333°E, 14.X.2003, D.Z. Dong leg. (IOZ); 3 males, 3 females, Dahaoping, along a small stream, 24°58'20.8"N, 98°44'20.1"E, 2170 m, 18.X.2003, H.B. Liang & X.C. Shi leg. (CAS, IOZ); 1 male, Shangying, 5–8 km E of Dahaoping, 2358 m, 24.93417°N, 98.74750°E, 18.X.2003, D.Z. Dong leg. (IOZ); 4 males, Shangying, 42-46 km on road from Bawan, 24°57'13.0"N, 98°44'32.1"E, 2290 m, 14.X.2003, H.B. Liang & X.C. Shi leg. (CAS, IOZ); 1 male, Shangying, Longjiang bridge, riverside, 25°02'29.7"N, 98°40'22.9"E, 1335 m, 19.X.2003, H.-B. Liang & S. Yang leg. (IOZ).

#### Distribution.

Fig. 58. This species was previously known from several localities, all restricted to the southern parts of the Gaoligong Shan in western Yunnan Province, China, at elevations of 2200–2400 m (Kataev and Schmidt 2006). The new material was also collected on the southern parts of Gaoligong Shan, at elevations of 1800-2500 m, within Longling, Longyang, and Tengchong counties, western Yunnan Province, China.

#### Habitat

**.** Specimens were collected in roadside and road cut open areas, on open, disturbed stream banks, and in other disturbed areas (Fig. 70), hidden under stones and other debris during daylight hours and active on the soil surface at night.

#### Remarks.

Kataev and Schmidt (2006) indicated the absence of the parascutellar setigerous pore on elytra in *Chydaeus convexus* as one of the distinctive features of this species belonging to the *semenowi* group. The examination of the addition material revealed the high variability of this character. Among 125 specimens examined, the parascutellar setigerous pore was absent from only 55 specimens, in a few from only one elytron.

### Chydaeus malaisei

Kataev & Schmidt, 2006

http://species-id.net/wiki/Chydaeus_malaisei

Fig. 59

#### Material examined.

 A total of 5 specimens (2 males and 3 females, including 1 male and 1 female in CAS and 1 male and 2 females in IOZ) were examined from the following localities: **China**. **Yunnan Province**. *Lushui County*: 1 male, Pianma, Ganheluo, riverside, 26.06210°N, 98.61966°E, 2100 m, 14.V.2005, H.B. Liang leg. (IOZ); 1 female, Pianma, Gangfang Yakou, road, 26.03672°N, 98.62026°E, 2250 m, 12.V.2005, H.B. Liang leg. (CAS); 1 female, Pianma, 6 km Pianma to Liuku, 26.00808°N, 98.65921°E, 2310 m, 15.V.2005, H.B. Liang leg. (IOZ); 1 female, Pianma, 6 km ESE Pianma, river, 26.00703°N, 98.16209°E, 2254 m, 15.V.2005, D.Z. Dong leg. (IOZ); 1 male, Pianma, Changyanhe, riverside, 25.99414°N, 98.66336°E, 2454 m, 15.V.2005, H.B. Liang leg. (CAS).

#### Distribution.

Fig. 59. This species was described from a series collected in Kambaiti, northeastern Myanmar. The new material from Yunnan was collected about 100 km to the northeast of the type locality, in the Pianma area, near the Myanmar border, on the western slope of the Gaoligong Shan, at elevations of 2100-2500 m. These new records support Kataev and Schmidt’s (2006) suggestion that the geographical range of *Chydaeus malaisei* is confined to the region west of the crest of the Gaoligong Shan.

#### Remarks.

 This species, a member of the *semenowi* group, is closely related to *Chydaeus convexus* and appears to be a geographical vicariant of the latter (Kataev and Schmidt 2006). The specimens examined from Yunnan demonstrate all the distinctive features of *Chydaeus malaisei* listed in the original description, except for the absence of the parascutellar setigerous pore on elytra. In all the specimens from Yunnan examined, this pore is present. As in the case for *Chydaeus convexus*, this character appears to be variable in *Chydaeus malaisei*.

### Chydaeus semenowi

(Tschitschérine, 1899)

http://species-id.net/wiki/Chydaeus_semenowi

Figs 59 71

#### Material examined.

 A total of 18 specimens (13 males and 5 females, including 5 males and 2 females in CAS, 7 males and 3 females in IOZ, and 1 male in ZIN) were examined from the following localities: **China**. **Xizang Autonomous Region**. *Nyalam County*:1 male, Xigaze, 3800 m, 6.VI.1961 (IOZ); 1 male, Nyalam, Zham, 3300 m, 7.VII.1975 (IOZ); 1 male, Nyalam, Zham, 3400 m, 7.VII.1975 (IOZ); + many additional specimens from Nyalam county (IOZ). **Yunnan Province**. *Gongshan County*: 2 males, 2 females, No 12 Bridge to Dulongjiang, 27°42'54"N, 98°30'08"E, 2770 m, 30.IV.2002, H.B. Liang, W. Ba & X. Li leg. (CAS, IOZ); 5 males, 1 female, same data, but 3.V.2002, H.B. Liang, W. Ba, G. Yang & L. Dou leg. (CAS, IOZ, ZIN); 1 male, Heiwadi, on new road to Dulongjiang, 27°47'39"N, 98°35'13"E, 2020 m, 20.IV.2002, H.B. Liang, W. Ba, G. Yang & X.Q. Li leg. (IOZ); 2 females, new road to Dulongjiang, 27°45'57"N, 98°36'12"E, 2200 m, 12.IV.2002, H.B. Liang & W. Ba leg. (CAS, IOZ); 1 female, Gaoligong Shan, Nujiang Pref., Danzhu He drainage, 13.5–15.7 air km SSW of Gongshan, 2700-3100 m, 27.63063°N, 98.62074°E to 27.62705°N, 98.59204°E, 30.VI.–5.VII.2000, Stop 00-17A, D. Kavanaugh, C.E. Griswold, H.B. Liang, D. Ubick & D.Z. Dong leg. (CAS); 1 male, Dulongjiang, Sandui, Bapo to Yakou, 27.71672°N, 98.42231°E, 1333 m, 30.X.2004, V.F. Lee leg. (CAS).

#### Distribution.

 According to Kataev and Schmidt (2006), *Chydaeus semenowi* is widely distributed over the Himalaya from Uttar Pradesh (India) to Bhutan, at elevations of 2400–3800 m. This species is recorded here from China (southern Xizang and northwestern Yunnan) for the first time (Fig. 59).

#### Habitat.

 Specimens were collected in roadside and road cut open areas, hidden under stones and other debris during daylight hours and active on the soil surface at night (Figs 71).

#### Remarks.

*Chydaeus semenowi*, a member of the *semenowi* group (Kataev and Schmidt 2006), is the most frequently encountered Himalayan species of *Chydaeus*. Specimens examined from southern Tibet are very similar in their morphology to other specimens from the Himalaya, but those from Yunnan are characterized by a smaller denticle at the apex of each pronotal basal angles, so they may represent a distinct geographical form.

### Chydaeus andrewesi

Schauberger, 1932

http://species-id.net/wiki/Chydaeus_andrewesi

#### Remarks.

Kataev and Schmidt (2006) treated *Chydaeus andrewesi* as a member of the *obscurus* group and recognized two subspecies: the nominotypic form, *Chydaeus andrewesi andrewesi*, distributed over the eastern Himalaya, and *Chydaeus andrewesi szetschuanus* Schauberger, 1932, occurring in Sichuan Province, China. Our study of additional material from China and Vietnam (see below) has convinced us that *Chydaeus kumei* Ito, 1992, described from Vietnam, is conspecific with *Chydaeus andrewesi* and represents a third subspecies of that species.

### Chydaeus andrewesi andrewesi

Schauberger, 1932

http://species-id.net/wiki/Chydaeus_andrewesi_andrewesi

Figs 60 68 –70 72

#### Material examined.

 A total of 358 specimens (183 males and 175 females, including 82 males and 82 females in CAS, 85 males and 82 females in IOZ, and 16 males and 11 females in ZIN and cBL&KB) were examined from the following localities: **China**. **Xizang Autonomous Region**. *Nyalam County*: 1 male, Nyalam, Zham, 1700 m, 22.VI.1975 (IOZ). **Yunnan Province**. *Fugong County*: 1 male, Pihe Town, Jianjiu Vill., roadside, 26.52842°N, 98.86997°E, 2132 m, 22.IV.2004, H.B. Liang leg. (IOZ); 1 female, Lumadeng, Laoshibali, roadside, 27.07978°N, 98.77328°E, 2305, 15.VIII.2005, D. Kavanaugh & D.Z. Dong leg. (IOZ); 1 male, Lumadeng, 4 km E Laoshibali, 27.09700°N, 98.80750°E, 2120 m, 21.VIII.2005, D.Z. Dong leg. (IOZ); 7 males, 1 female, Lumadeng, Yanping-Shibali, 11 km, 27.13839°N, 98.82147°E, 1850 m, 25.IV.2004, H.B. Liang leg. (CAS, IOZ); 2 males, 2 females, Lumadeng, Yanping-Shibali, roadside, 27.14627°N, 98.81559°E, 2030 m, 3.V.2004, H.B. Liang & M. Xie leg. (CAS, IOZ); 1 male, 1 female, Lumadeng, Yaping, Yamuhe, roadside, 27.11876°N, 98.83118°E, 1800 m, 26.IV.2004, H.B. Liang leg. (IOZ); 2 males, 2 females, Lumadeng, Yaping, Rimalige, road, 27.09728°N, 98.80475°E, 2040 m, 4.V.2004, H.B. Liang & B.-X. Zhu leg. (CAS, IOZ); 2 males, Lumadeng, Yaping, Yejiadi, roadside, 27.08004°N, 98.77325°E, 2307 m, 10.V.2004, H.B. Liang & B.-X. Zhu leg. (CAS, IOZ); 1 male, Lumadeng, 4 km up Yaping Bridge, 27.12817°N, 98.85944°E, 1500 m, 11.VIII.2005, H.B. Liang & J.F. Zhang leg. (IOZ); 1 male, 1 female, Lumadeng Town, Aludi Vill., Nujiang, 27.09830°N, 98.87272°E, 1195 m, 23.IV.2004, D. Kavanaugh leg. (IOZ); 4 males, 5 females, Lumadeng Town, Shilajia on Yaping road, 27.13419°N, 98.82641°E, 1800 m, 24–25.IV.2004, D. Kavanaugh leg. (CAS, IOZ); 1 female, Lumadeng Town, Yaping road above Shilajia, 27.13086°N, 98.83874°E, 1630 m, 26.IV.2004, D. Kavanaugh & C. Griswold leg. (IOZ); 1 female, Maji Town, Majimi Vill., riverside, 27.39630°N, 98.81701°E, 1567 m, 28.IV.2004, H.B. Liang leg. (IOZ); 1 female, Lishadi Town, Shibali, 1.5 km down road, 27.16284°N, 98.78989°E, 2420 m, 2.V.2004, H.B. Liang & G.-X. Peng leg. (IOZ); 1 male [teneral], Lishadi Town, 4 km below Shibali, road, 27.15727°N, 98.79784°E, 2280 m, 11.VIII.2005, H.B. Liang leg. (IOZ). *Gongshan County*: 1 male, 1 female, Cikai Town, Dandang Park, roadside, 27.74853°N, 98.66492°E, 1605 m, 23.X.2004, V.F. Lee leg. (IOZ); 1 male, Cikai Town, Pulahe, river & road side, 27°44'55"N, 98°40'01E, 1445 m, 8.X.2002, D. Kavanaugh & H.B. Liang leg. (CAS); 1 female, Cikai, Pulahe, Pulahe Power Station dam, 27.76305°N, 98.62540°E, 1605 m, 23.X.2004, H.B. Liang leg. (IOZ); 1 male, Cikai Town, Pulahe, joint with Nujiang, 27.74843°N, 98.66498°E, 1530 m, 11.XI.2004, D. Kavanaugh & D.Z. Dong leg. (IOZ); 2 males, Cikai Township, 16.8 km of Gongshan on Dulong valley Road at Heiwadi, 2150 m, 10.X.2002, Stop DHK-2002-044E, D. Kavanaugh, P.E. Marek, H.B. Liang & D.-Z. Dong leg. (CAS, IOZ); 1 male, Cikai Town - Qiqi Station, 27.43086°N, 98.34150°E, 1700–2000 m, 29.IV.2002, H.B. Liang & W. Ba leg. (IOZ); 1 female, Cikai Town, Gazu Station, 27°44'35"N, 98°36'17"E, 1500 m, 4.V.2002, H.B. Liang & W. Ba leg. (IOZ); 2 males, Cikai Town, Cikaihe, 27°43'59"N, 98°39'32"E, 1730 m, 22.IV.2002, H.B. Liang, W. Ba, X. Li & G.D. Yang leg. (IOZ); 3 males, 4 females, Cikai Town, along street, 27°44'43"N, 98°39'53'E, 1500 m, 13.IV.2002, H.B. Liang & W. Ba leg. (CAS, IOZ); 3 males, 1 female, same data, but 20.IV.2002 (CAS, IOZ); 2 males, Cikai Town, Heiwadi, road side, 27°47'40"N, 98°35'21"E, 2010 m, 10.X.2002, H.B. Liang leg. (IOZ); 2 males, 1 female, Heiwadi, on new road to Dulongjiang, 27°47'39"N, 98°35'13"E, 2020 m, 20.IV.2002, H.B. Liang & W. Ba leg. (IOZ); 3 males, 2 females, same data, but H.B. Liang, W. Ba, G. Yang & X.Q. Li leg. (CAS, IOZ); 2 males, 3 females, Heiwadi, night, 27°47'39"N, 98°35'13"E, 2030 m, 22.IV.2002, H.B. Liang, W. Ba, X. Li & G.D. Yang leg. (CAS, IOZ); 1 female, Cikai Town, Heiwadi Dabadi, 27°45'24"N, 98°34'56"E, 2470 m, 10.X.2002, X. Li leg. (IOZ); 1 male, Shunglawa-Cilou, 27°46'14"N, 98°39'16"E, 1650 m, 5.V.2002, H.B. Liang, W. Ba, G. Yang & X.Q. Li leg. (IOZ); 1 male, Cilou (Power Station), 27°46'14"N, 98°39'16"E, 1510 m, 6.V.2002, H.B. Liang, W. Ba, G. Yang & X.Q. Li leg. (IOZ); 3 males, 5 females, Yeniugu, along road, 27°43'3"N, 98°44'14"E, 2020 m, 16.IV.2002, H.B. Liang & W. Ba leg. (CAS, IOZ); 2 female, new road to Dulongjiang, 27°45'57"N, 98°36'12"E, 2200 m, 12.IV.2002, H.B. Liang & W. Ba leg. (CAS, IOZ); 1 female, Dulongjiang, Qinlangdang, 27.69033°N, 98.27901°E, 1300 m, 31.VIII.2006, D.Z. Dong leg. (IOZ); 20 males, 30 females, Dulongjiang, 0.6 km N Dizhengdang, 28.08442°N, 98.32652°E, 1880 m, 29.X.2004, D. Kavanaugh leg. (CAS, IOZ); 1 female, same data, but 30.X.2004, D. Kavanaugh & D.Z. Dong leg. (IOZ); 1 male, 4 females, Dulongjiang, Dizhengdang, Silalong He, 28.07654°N, 98.32603°E, 1890 m, 30.X.2004, D. Kavanaugh & D.Z. Dong leg. (CAS, IOZ); 1 male, Dulongjiang, 2.8 km S Longyuan Vill., 28.00905°N, 98.32204°E, 1660 m, 31.X.2004, D. Kavanaugh & D.Z. Dong leg. (IOZ); 1 female, Dulongjiang, 2.3 km S Longyuan Vill., 28.00532°N, 98.32145°E, 1685 m, 2.XI.2004, D. Kavanaugh leg. (IOZ); 2 males (1teneral), Dulongjiang, Elidang Village, beach, 28.00287°N, 98.32145°E, 1640 m, 3.XI.2004,D. Kavanaugh & D.Z. Dong leg. (CAS, IOZ); 1 male, Dulongjiang, Xianjiudang Village, 27.94092°N, 98.33340°E, 1880 m, 4.XI.2004, D. Kavanaugh & D.Z. Dong leg. (IOZ); 1 male, 2 females, Dulongjiang, Pengjiawang, above Bapo, 27.72999°N, 98.40650°E, 2250 m, 28.X.2004, H.B. Liang. (IOZ); 1 male, Dulongjiang, Penjiasheng, above Bapo, 27.73053°N, 98.40561°E, 2233 m, 31.X.2004, V.F. Lee leg. (IOZ); 1 male, Dulongjiang, 0.5 km N Kongdang, 27.88111°N, 98.34062°E, 1500 m, 7.XI.2004, D. Kavanaugh leg. (IOZ); 4 males, 8 females, Dulongjiang, Maku Vill., roadside, 27.68533°N, 98.30425°E, 1823 m, 1–2.XI.2004, H.B. Liang leg. (CAS, IOZ); 1 male, Dulongjian, 0.5 km WSW Maku, trail, 27.68310°N, 98.30038°E, 1845 m, 29.VIII.2006, D. Kavanaugh leg. (IOZ); 1 male, 1 female, Dulongjiang, Maku Vill., 27.68545°N, 98.30419°E, 1814 m, 29.VIII.2006, D.Z. Dong & P.Hu leg. (IOZ); 1 female, same data, but 27.68804°N, 98.30758°E, 1615 m, 3.IX.2006, D. Kavanaugh & Y. Liu leg. (IOZ); 1 male, Maxidang, along road, 27°52'41"N, 98°39'15"E, 1550 m, 19.IV.2002, H.B. Liang, W. Ba, G. Yang & X.Q. Li leg. (IOZ); 30 males, 23 females, Bingzhongluo, Gongdangshen Shan, 27°59'51"N, 98°37'7"E, 2480 m, 24.IV.2002, H.B. Liang, W. Ba, G. Yang & X.Q. Li leg. (CAS, IOZ, ZIN); 1 male, Bingzhongluo, Gongdangshen Shan, 27.99725°N, 98.62003°E, 2489 m, 12.XI.2004, H.B. Liang leg. (IOZ); 15 males, 15 females, Bingzhonluo vill., 1700 m, 26.IV.2002, H.B. Liang & W. Ba leg. (CAS, IOZ); 1 female, Bingzhongluo, Yimaluo, riverside, 28°01'30"N, 98°37'33"E, 1606 m, 8.X.2002, H.B. Liang leg. (IOZ); 1 male, Bingzhongluo, Niwaluo, under rocks, tree, 28.03287°N, 98.56995°E, 1862 m, 15.VIII.2006, Y. Liu & P. Hu leg. (IOZ). *Longyang County* (*District*): 1 female, Bawan, 36–37 km on old road to Tengchong, 24°56'03.3"N, 98°46'46.4"E, 2150 m, 12.X.2003, H.B. Liang & X.C. Shi leg. (IOZ); 1 male, 2 females, Bawan Town, Nankang forest station, 24°49'28.8"N, 98°46'43.6"E, 2085 m, 27.X.2003, H.B. Liang & X.C. Shi leg. (CAS, IOZ); 1 female, Bawan, Nankang station, 24.82260°N, 98.78201°E, 2060 m, 23.V.2005, H.B. Liang leg. (IOZ); 1 male, 1 female, same data, but 2048 m, 22.V.2005, H.B. Liang leg. (IOZ); 2 males, 1 female, Bawan Township, Nankang Yakou, 24.81944°N, 98.77111°E, 2130 m, 31.X.2003, under rocks, D.Z. Dong leg. (CAS); 2 females, same data, but 24.82583°N, 98.77222°E, 2130 m, 26.X.2003 (CAS, IOZ); 1 female, Bawan subdist., 34 km from Bawan on Tengchong Road, 2310 m, 24.92944°N, 98.75917°E, 16.X.2003, under rocks, D.Z. Dong leg. (IOZ). *Lushui County*: 1 male, 2 females, Pianma, 6 km Pianma to Liuku, 26.00808°N, 98.65921°E, 2310 m, 15.V.H.B. Liang leg. (IOZ); 2 males, 1 female, Pianma, 6 km ESE Pianma, river, 26.00703°N, 98.16209°E, 2254 m, 15.V.2005, D. Dong leg. (IOZ); 1 female, Pianma, Xiapianma, roadside, 26.00992°N, 98.61670°E, 1780 m, 15.V.2005, D. Kavanaugh & D. Dong leg. (IOZ); 1 male, 1 female, same data, but 26.00950°N, 98.61704°E, 1780 m, riverside, 15.V.2005, H.B. Liang leg. (IOZ); 1 female, same data, but H.B. Liang leg. (IOZ); 1 female, Pianma, Gangfang, roadside, 26.11781°N, 98.59342°E, 1787 m, 16.V.2005, H.B. Liang & Y.H. San leg. (IOZ); 4 males, 1 female, Pianma, Gangfang, Xuetang, 26.12218°N, 98.57546°E, 1625 m, 16.V.2005, D. Kavanaugh & D.Z. Dong leg. (IOZ, CAS); 1 male, 7 females, Pianma, Changyanhe, riverside, 25.99414°N, 98.66336°E, 2540 m, 12.V.2005, D. Kavanaugh & D. Dong. (CAS, IOZ); 1 female, Pianma, Gulangba, roadside, 26.11253°N, 98.06250°E, 1563 m, 14.V.2005, D.Z. Dong leg. (IOZ); 1 male, 2 females, Luzhang, Langbazhai, Lusaihe, 25.96567°N, 98.77091°E, 1820 m, 20.V.2005, D.Z. Dong leg. (IOZ). *Shuangjiang County*: 1 male, Mt. NW Mengku Town, 23°40'28"N, 99°48'11"E, 23°40'29"N, 99°46'53"E, 2125–2720 m, 26.V.2010, I. Belousov & I. Kabak leg. (cBL&KB). *Tengchong County*: 1 male, Dahaoping, 46-51 km on old road, 24°57'25.6"N, 98°44'12.3"E, 2220 m, 17.X.2003, H.B. Liang & X.C. Shi leg. (IOZ); 1 female, Jietou, Datang, Dahelingganjiao, 25.73939°N, 98.69633°E, 2010 m, 19.V.2006, H.B. Liang & Z.C. Liu leg. (IOZ); 5 males, 2 females, same data, but 25.73947°N, 98.69630°E, 2010 m, 14-15.V.2006, D. Kavanaugh & R. Brett leg. (CAS, IOZ); 1 female, same data, but 19.V.2006 (IOZ); 2 males, same data, but 25.73678°N, 98.69639°E, 2005 m, 18.V.2006, D.Z. Dong leg. (CAS, IOZ); 1 male, 1 female, same data, but 25.73947°N, 98.69630°E, 2010 m, 18.V.2006, D. Kavanaugh & R. Brett leg. (CAS); 3 males, 1 female, same data, but 16.V.2006, D. Kavanaugh & R. Brett leg. (CAS, IOZ); 2 males, same data, but 14.V.2006, D.Z. Dong & X.P. Wang leg. (CAS, IOZ); 1 female, same data, but 25.75523°N, 98.69305°E, 1970 m, 16.V.2006, H.B. Liang leg. (IOZ); 2 males, Jietou, Datang, Dahetou, 25.69700°N, 98.68059°E, 1865 m, 14.V.2006, H.B. Liang leg. (CAS, IOZ); 1 female, Houqiao, Gaoshidong, roadside, 25.39858°N, 98.30533°E, 2580 m, 27.V.2006, D. Kavanaugh & R. Brett leg. (ZIN); 1 male, Houqiao, Guyong Linchang, ground, 25.36538°N, 98.32412°E, 2950 m, 27.V.2006, H.B. Liang & Z.C. Liu leg. (IOZ). *Xinping County*: 1 male, Ailaoshan Mt. Range, W Shuitangzhen Town, 24°08'07.1"N, 101°26'17"E, 2300 m, 1.VI.2011, I. Belousov, I. Kabak & A. Korolev leg. (cBL&KB); 3 males, same data, but 24°08'24"N, 101°25'16"E, 1940 m, 2.VI.2011, I. Belousov, I. Kabak & A. Korolev leg. (cBL&KB); 3 males, Ailaoshan Mt. Range, NW Shuitangzhen Town, 24°09'51"N, 101°25'29"E, 2005 m, 7.VI.2011, I. Belousov, I. Kabak & A. Korolev leg. (cBL&KB, ZIN); 6 males, 9 females, SSE Shuangjiang Town, 23°23'19"N, 99°55'28"E, 2255 m, 21.VI.2011, I. Belousov, I. Kabak & A. Korolev leg. (cBL&KB, ZIN); 1 male, same data, but 23°22'22"N, 99°54'47"E, 2540 m, 22.VI.2011, I. Belousov, I. Kabak & A. Korolev leg. (cBL&KB).

#### Distribution.

Fig. 60. The nominotypic subspecies of *Chydaeus andrewesi* was known previously from the eastern part of the Himalaya, from Central Nepal to Bhutan and Myanmar, at elevations of 1600–2700 m (Kataev and Schmidt 2006). Based on the new records presented here, this subspecies ranges farther east into western Yunnan Province, China, along the Myanmar border (the Gaoligong Shan in Fugong, Gongshan, Longyang, Lushui, and Tengchong counties, and the Bangma Shan and Ailao Shan (Mountains), at elevations of 1200–2720 m. This subspecies is also recorded here from the southern part of Xizang Autonomous Region.

#### Habitat.

 Specimens were collected in roadside and road cut open areas (Figs 68, 72), on open, disturbed stream banks, and in other disturbed areas (Figs 69–70), hidden under stones and other debris during daylight hours and active on the soil surface at night.

#### Remarks.

 This is the first record of *Chydaeus andrewesi andrewesi* from China. There are no significant morphological differences between the specimens from China and those from the areas in the western part of its geographical range; however, specimens from Yunnan have the elytral microsculpture more fully effaced than specimens from the Himalaya.

### Chydaeus andrewesi kumei

Ito, 1992 stat. n.

http://species-id.net/wiki/Chydaeus_andrewesi_kumei

Figs 47–50 60 74

Chydaeus kumei Ito, 1992: 52
? Chydaeus (Chydaeus) guangxiensis Ito, 2006: 198

#### Material examined.

 A total of 35 specimens (25 males and 10 females, including 1 male in IOZ and 24 males and 10 females in cFED, cSCH, cWR, and ZIN) were examined from the following localities: **China**. **Yunnan Province**. *Jinping County*: 1 male, Fenshuiling, Leidazhan, roadside, 22°51'41.0"N, 103°13'40.5"E, 2060 m, 17.XII.2003, H.B. Liang leg. (IOZ). **Vietnam**. **Lao Cai Province**: 2 males, 2 females, 6 km W of Sa Pa, N slope of Phansipan Mt. Area, 2000–2100 m, near Tram don (base of Hoang Lien Nature Park), 22°21'N, 103°46'E, V.2005, A.V. Abramov leg. (Exp. of Russia-Vietnam Tropical Centre) (ZIN); 1 female, same data, but 1930-2000 m, V.2010, A.V. Abramov leg. (ZIN); 18 males, 6 females, 6 km W of Sa Pa, northern slope of Phansipan Mt. area, May 2008, A.V. Abramov leg. (Exp. of Russia-Vietnam Tropical Centre) (cSCH, cWR, ZIN); 4 males, 1 female, Sa Pa env., ca 1600 m, V.2006, A. Anitchkin leg. (cFED).

#### Distribution.

Fig. 60. Previously, this taxon was known only from the single male collected in Sa Pa, northern Vietnam. Based on the new records presented here, this species occurs not only in the mountains of northern Vietnam, but also in southern Yunnan Province (China) adjacent to the Vietnamese border.

#### Habitat.

 Specimens were collected in roadside and road cut open areas and other disturbed areas adjacent to moderately disturbed forest (Fig. 74), hidden under stones and other debris during daylight hours.

#### Remarks.

**Figures 47–50.**
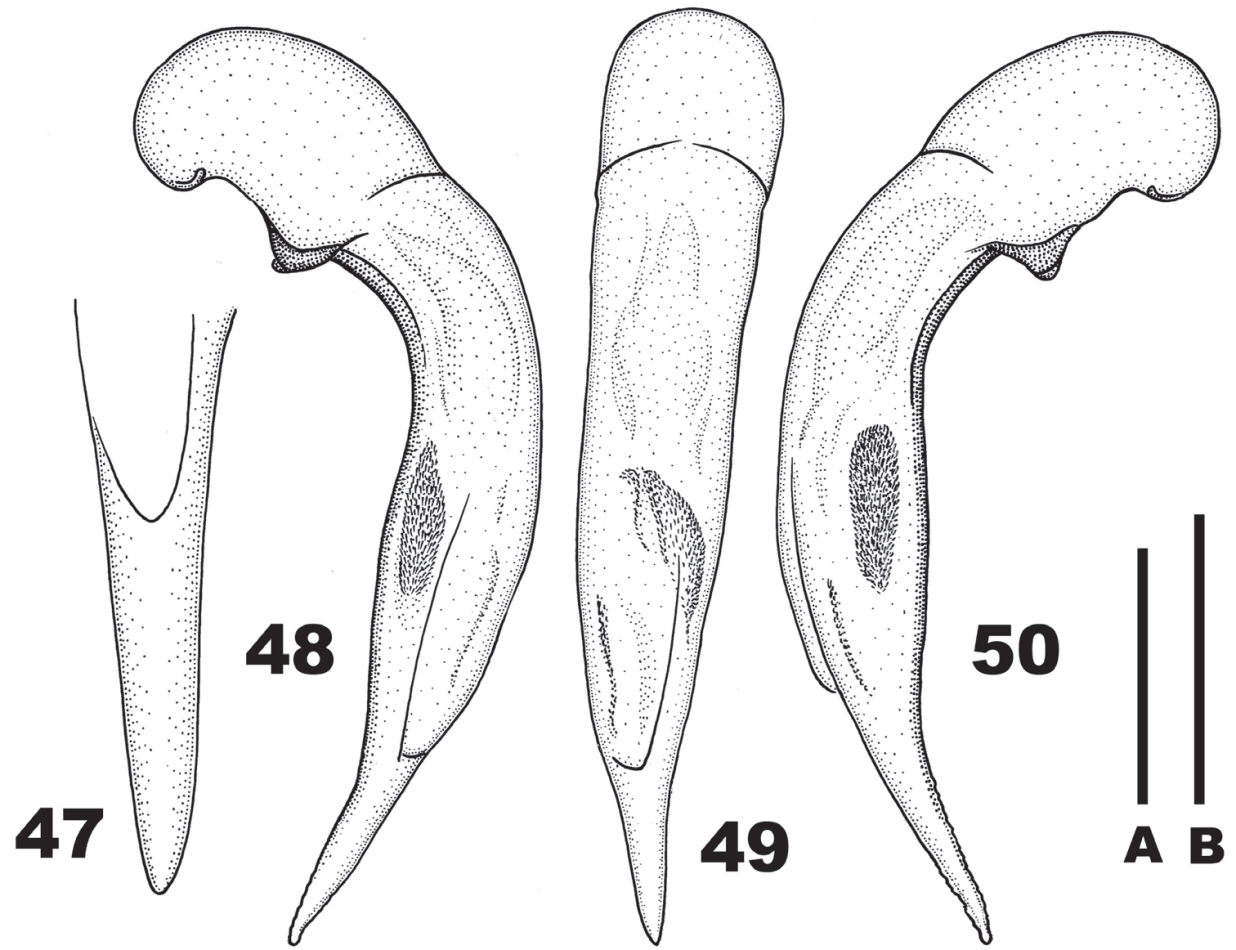
*Chydaeus andrewesi kumei* Ito (Vietnam, Sa Pa area) **47** Terminal lamella of median lobe, dorsal view **48** Median lobe, left lateral view **49** Median lobe, dorsal view. **50** Median lobe, right lateral view. Scale lines: **A** = 0.5 mm (Fig. 47), **B** = 1.0 mm (Figs 48–50).

 Based on Ito’s original description, Kataev and Schmidt (2006) treated *Chydaeus kumei* as a distinct species of the *obscurus* group, closely related to *Chydaeus andrewesi* as characterized by Ito (1992). Examination of specimens of *Chydaeus kumei* revealed that this taxon possesses all the distinctive features of *Chydaeus andrewesi* listed by Kataev and Schmidt (2006), including the elongate metepisterna (in both taxa, their length along inner margin is much greater than the width along the anterior margin). Moreover, the structure of the aedeagus in *Chydaeus kumei* males (Figs 47–50) is virtually identical to that in *Chydaeus andrewesi* males and, in our opinion, *Chydaeus kumei* should be treated as a subspecies of *Chydaeus andrewesi*. The vicariant distributions of these taxa in Yunnan also supports the subspecific status of *Chydaeus kumei*.

The main distinctive characters of adults of *Chydaeus andrewesi kumei* are: pronotum markedly narrowed basad, distinctly depressed and comparatively coarsely punctate laterobasally; pronotal basal angles each with a denticulate apex protruded laterad; elytral disc in males with very fine, more or less markedly effaced microsculpture comprised of thin, transverse meshes; proepisterna distinctly punctate anteriorly; and base of the terminal lamella of the median lobe of aedeagus (Fig. 47) slightly wider than that of other subspecies. We add the following mensural features to Ito’s description: Size: Body length 8.6–10.2 mm, width 3.8–4.5 mm. Proportions: HWmax/PWmax = 0.71–0.74; HWmin/PWmax = 0.59-0.63; PWmax/PL = 1.41–1.48; PWmax/ PWmin = 1.26–1.40; EL/EW = 1.38–1.43, EL/PL = 2.39-2.59 (2.39–2.51 in male and 2.42–2.59 in female), W/PWmax = 1.19–1.29.

Specimens of *Chydaeus andrewesi kumei* are very similar to those of *Chydaeus andrewesi szetschuanus* from Sichuan in morphological features, particularly in the shape of the pronotum (with denticulate basal angles), the punctate proepisterna, and the relatively wide elytra; but they differ from the latter mainly in having finer and sparser punctation on the apical half of the pronotum (pronotum densely and coarsely punctate anteriorly in *Chydaeus andrewesi szetschuanus* specimens).

*Chydaeus guangxiensis* Ito, 2006 was described from one male from Guangxi ("Below Tienshan Ping, Mt. Miao'er, Xing'an Xian”). We examined two females from the same mountain [one labeled: “Guangxi, Mt. Miao’er, 2000m, 1985.VIII.1, Song Shimei leg. (IOZ); for the other, see Kataev and Schmidt (2006: 145)], that matched the original description of *Chydaeus guangxiensis* very well. Judging from Ito's original drawings (2006: Fig. 4), the aedeagus of *Chydaeus guangxien*sis males is identical to that of *Chydaeus andrewesi* males. The pronotum of *Chydaeus guangxien*sis adults is smooth or finely punctate anteriorly, as in *Chydaeus andrewesi kumei* adults, but the denticle at each basal pronotal angle is only slightly evident or absent, as in *Chydaeus andrewesi andrewesi* specimens. Further study, based on additional specimens, is necessary to determine the taxonomic status of *Chydaeus guangxien*sis; but it is very likely that this taxon is either consubspecific with *Chydaeus andrewesi kumei* or, at most, another subspecies of *Chydaeus andrewesi*.

**Figures 51–55.**
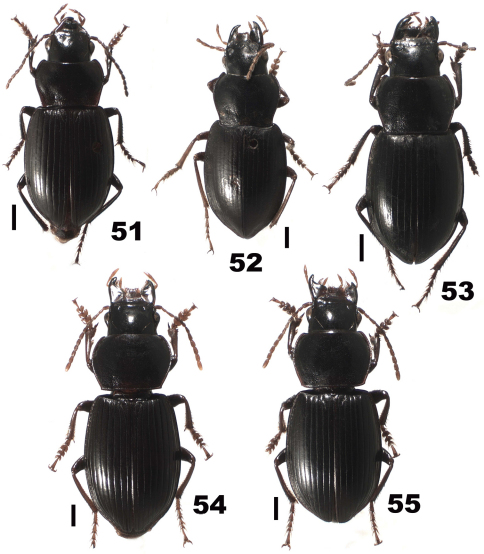
Dorsal habitus, *Chydaeus* species. **51** *Chydaeus fugongensis* sp. n. (holotype). **52** *Chydaeus gutangensis* sp. n. (holotype). **53** *Chydaeus hanmiensis* sp. n. (holotype). **54** *Chydaeus asetosus* sp. n. (holotype) **55** *Chydaeus baoshanensis* sp. n. (holotype). Scale lines = 1.0 mm.

**Figures 56–57.**
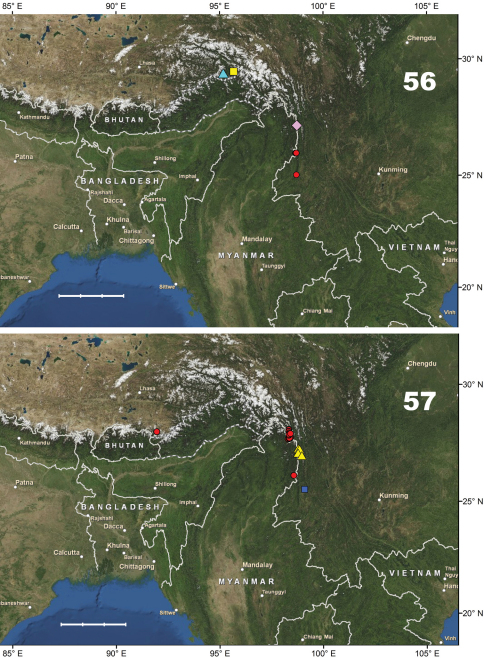
Toporelief map of southeastern Asia, illustrating localities for *Chydaeus* species **56** *Chydaeus shunichii* Ito = red dots, *Chydaeus fugongensis* sp. n. = pink diamond, *Chydaeus gutangensis* sp. n. = yellow square, and *Chydaeus hanmiensis* sp. n. = light blue triangle **57** *Chydaeus baoshanensis* sp. n. = dark blue square, *Chydaeus asetosus* sp. n. = yellow triangles, and *Chydaeus obtusicollis* Schauberger = red dots. Only new records reported here are shown. Scale bar = 300 km (in 100 km increments).

**Figures 58–59.**
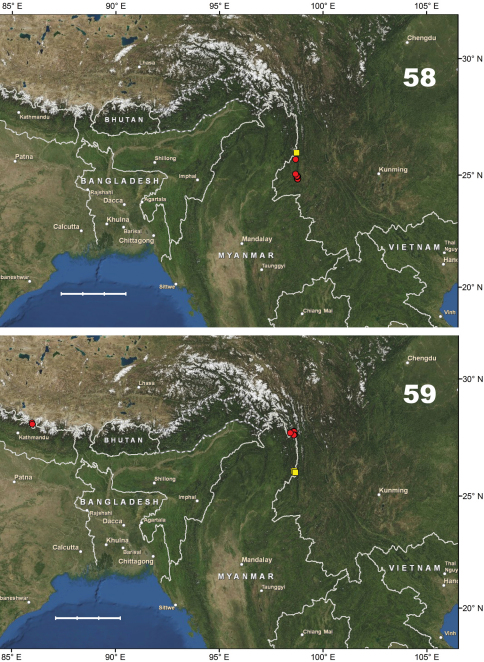
Toporelief map of southeastern Asia, illustrating localities for *Chydaeus* species **58** *Chydaeus satoi* Ito = yellow square and *Chydaeus convexus* Ito = red dots **59** *Chydaeus semenowi* (Tschitschérine) = red dots and *Chydaeus malaisei* Kataev & Schmidt = yellow squares. Only new records reported here are shown. Scale bar = 300 km (in 100 km increments).

**Figures 60–61.**
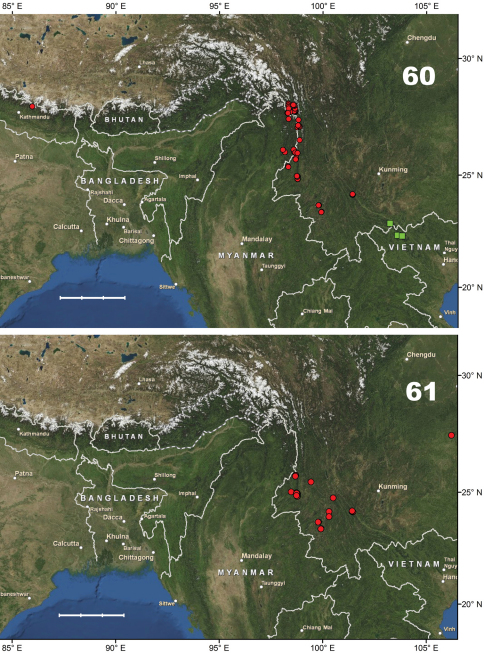
Toporelief map of southeastern Asia, illustrating localities for *Chydaeus* species **60** *Chydaeus andrewsi* Schauberger: *Chydaeus andrewsi andrewsi* Schauberger = red dots and *Chydaeus andrewsi kumei* Ito = green squares **61** *Chydaeus salvazae Schauberger* = red dots. Only new records reported here are shown. Scale bar = 300 km (in 100 km increments).

### Chydaeus salvazae

Schauberger, 1934a

http://species-id.net/wiki/Chydaeus_salvazae

Figs 61 70

= Chydaeus (Chydaeus) nigricans Ito, 2002: 300.
= Chydaeus oblongulus Ito, 2003: 83, nomen nudum, NEW STATUS

#### Material examined.

 A total of 542 specimens (297 males and 245 females, including 110 males and 107 females in CAS, 114 males and 109 females in IOZ, and 73 males and 29 females in cBL&KB and ZIN) were examined from the following localities: **China**. **Guizhou Province**. *Chishui County*: 1 male, 2 females, Jinsha Vill., 500 m, 21.IX.2000, H.B. Liang leg. (IOZ). **Yunnan Province**. *Longling County*: 1 female, Longjiang, Xiaoheishan, roadside, 24.82888°N, 98.76001°E, 2020 m, 2005.V.25, D. Kavanaugh & D.Z. Dong leg. (CAS); 1 female, same data, but riverside, 24.82886°N, 98.75917°E, 2010 m, H.B. Liang leg. (IOZ). *Longyang County* (*District*): 58 males, 43 females, Bawan Town, Nankang forest station, 24°49'28.8"N, 98°46'43.6"E, 2085 m, 27.X.2003, H.B. Liang & X.C. Shi leg. (CAS, IOZ, ZIN); 1 male, Bawan, Nankang station, 24.82600°N, 98.77690°E, 2090 m, 28.V.2005, D. Kavanaugh leg. (ACS); 11 females, same data, but 24.82614°N, 98.77602°E, 1900 m, 26.V.2005, D.Z. Dong leg. (CAS, IOZ); 2 males, 1 female, same data, but 24.83178°N, 98.76462°E, 2180 m, 22.V.2005, D. Kavanaugh & D.Z. Dong leg. (CAS, IOZ); 54 males, 32 females, Bawan, Nankang station, 24.82284°N, 98.78207°E, 2060 m, 23.V.2005, D. Kavanaugh & D.Z. Dong leg. (CAS, IOZ); 4 males, 3 females, same data, but 24.82600°N, 98.77690°E, 2090 m, 28.V.2005, D. Kavanaugh leg. (CAS, IOZ); 36 males, 21 females, same data, but 24.82587°N, 98.76832°E, 2048 m, 22.V.2005, H.B. Liang leg. (CAS, IOZ); 20 males, 55 females, Bawan Town, Nankang Yakou, roadside, 24°49'33.4"N, 98°46'20.0"E, 2130 m, 26.X.2003, H.B. Liang & X.C. Shi leg. (IOZ); 11 males, 10 females, same data, but 24.81944°N, 98.77111°E, 2130 m, 31.X.2003, D.Z. Dong leg. (IOZ); 1 male, 6 females, same data, but 27.X.2003 (CAS, IOZ); 13 males, 25 females, same data, but 24.82583°N, 98.77222°E, 2130 m, D.Z. Dong leg. (CAS, IOZ); 3 males, 6 females, Bawan, Dasheyao forest station - Yakou, 24°55'37.4"N, 98°45'09.8"E, 2404 m, 12.X.2003, H.B. Liang & X.C. Shi leg. (IOZ); 1 female, Bawan, Dasheyao, 24.92989°N, 98.75862°E, 2320 m, 3.VI.2005, J. Yang leg. (IOZ); 1 male, Bawan, 36–37 km on old road to Tenchong, 24°56'03.3"N, 98°46'46.4"E, 2150 m, 12.X.2003, H.B. Liang & X.C. Shi leg. (IOZ); 1 male, Bawan, 35 km on old road to Tengchong, 24°56'01.5"N, 98°47'04.1"E, 2010 m, 16.X.2003, H.B. Liang & J.J. Yang leg. (IOZ); 1 male, 1 female, Bawan, Luokeng, 41 km on road to Tengchong, 24°56'23.2"N, 98°45'11.6"E, 2440 m, 15.X.2003, H.B. Liang & X.C. Shi leg. (IOZ). *Nanjian County*: 3 males, Wuliangshan Mt. Range, 24°45'02"N, 100°30'24"E, 2270 m, 12.VI.2011, I. Belousov, I. Kabak & A. Korolev leg. (cBL&KB, ZIN). *Shuangjiang County*: 1 female, Mt. NW Mengku Town, 23°40'28"N, 99°48'11"E, 23°40'29"N, 99°46'53"E, 2125–2720 m, 26.V.2010, Belousov & Kabak leg. (cBL&KB). *Tengchong County*: 1 male, Jietou, Datang, Dahelingganjiao, 25.73947°N, 98.69630°E, 2010 m, 18.V.2006, D. Kavanaugh & R. Brett leg. (IOZ); 1 male, same data, but 19.V.2005 (CAS); 1 female, same data, but 14.V.2006, D.Z. Dong & X.P. Wang leg. (IOZ); 1 male, same data, but 25.72717°N, 98.69322°E, 1960 m, 19.V.2006, D. Kavanaugh & R. Brett leg. (CAS); 1 female, same data, but 25.69700°N, 98.68059°E, 1800–2000 m, 16.VI.2005, H. Huang leg. (IOZ); 1 male, Dahaoping, 46–51 km on old road, 24°57'25.6"N, 98°44'12.3"E, 2220 m, 17.X.2003, H.B. Liang & X.C. Shi leg. (IOZ); 4 males, 2 females, Dahaoping, along a small stream, 24°58'20.8"N, 98°44'20.1"E, 2170 m, 18.X.2003, H.B. Liang & X.C. Shi leg. (CAS, IOZ); 1 male, Dahaoping, Forest station, roadside, 24°58'31.8"N, 98°43'47.8"E, 2014 m, 18.X.2003, Tang Guo et al. (IOZ); 1female, same data, but 24.96942°N, 98.73472°E, 2072 m, 31.V.2005, D.Z. Dong leg. (IOZ); 3 males, Shangying, Dahaoping Station, 24.96976°N, 98.73142°E, 2040 m, 31.V.2005, H.B. Liang leg. (CAS, IOZ); 4 males, Tengyue, Laifengshan, headlamp, 25.01734°N, 98.47719°E, 1920 m, 1.VI.2006, D. Kavanaugh leg. (CAS, IOZ); 2 females, Dahaoping, 46–51 km on old road, 24°57'25.6"N, 98°44'12.3"E, 2220 m, 17.X.2003, H.B. Liang & X.C. Shi leg. (CAS, IOZ); 1 female, Shangying, 42-46 km on road from Bawan, 24°57'13.0"N, 98°44'32.1"E, 2290 m, 14.X.2003, H.B. Liang & X.C. Shi leg. (IOZ); 1 male, Dahaoping, 46-51 km on old road, 24°57'25.6"N, 98°44'12.3"E, 2220 m, 17.X.2003, H.B. Liang & X.C. Shi leg. (IOZ); 1 male, 1 female, Wuhe, Zhengding Forest Station, 24.85458°N, 98.73743°E, 1828 m, 26.V.2005, H.B. Liang leg. (IOZ). *Xinping County*: 2 males, Ailaoshan Mt. Range, W Shuitangzhen Town, 24°07'18"N, 101°27'44"E, 1965 m, 31.V.2011, I. Belousov, I. Kabak & A. Korolev leg. (cBL&KB); 4 males, 2 females, same data, but 24°08'07"N, 101°26'17"E, 2300 m, 1.VI.2011, I. Belousov, I. Kabak & A. Korolev leg. (cBL&KB, ZIN); 3 males, 1 female, same data, but 24°08'24"N, 101°25'16"E, 1940 m, 2.VI.2011, I. Belousov, I. Kabak & A. Korolev leg. (cBL&KB); 2 males, 1 female, Ailaoshan Mt. Range, NW Shuitangzhen Town, 24°09'51"N, 101°25'29"E, 2005 m, 7.VI.2011, I. Belousov, I. Kabak & A. Korolev leg. (cBL&KB, ZIN); 56 males, 21 females, SSE Shuangjiang Town, 23°23'19"N, 99°55'28"E, 2255 m, 21.VI.2011, I. Belousov, I. Kabak & A. Korolev leg. (cBL&KB, ZIN); 1 female, same data, but 23°22'22"N, 99°54'47"E, 2540 m, 22.VI.2011, I. Belousov, I. Kabak & A. Korolev leg. (cBL&KB); 2 males, ENE Lincang Town, 23°54'58"N, 100°18'33"E, 2190 m, 25.VI.2011, I. Belousov, I. Kabak & A. Korolev leg. (cBL&KB, ZIN). *Yongping County*: 1 female, Bonan, Zhuopan Vill., 2000 m, 25.44507°N, 99.43715°E, 22.VIII.2007, B. Kataev & H.B. Liang leg. (ZIN).

#### Distribution.

*Chydaeus salvazae* is distributed over northern Vietnam, southwestern China (Guizhou, Sichuan, and Yunnan provinces) and the Central Himalaya (Sikkim and Nepal) (Kataev and Schmidt 2006). In Yunnan, the species is common in southern (Pingbian and Jinping counties) and western (Longling, Longyang, Shuangjiang, Tengchong, and Yongping counties) parts of the province (Fig. 61).

#### Habitat.

 Specimens were collected in roadside and road cut open areas, on open, disturbed stream banks, and in other disturbed areas (Fig. 70), hidden under stones and other debris during daylight hours and active on the soil surface at night.

#### Remarks.

 This species belongs to the monobasic *salvazae* group (Kataev and Schmidt 2006). Among the species of *Chydaeus* occurring in Yunnan, Ito (2003: 83) listed the name of *Chydaeus oblongulus* but omitted his recently described species *Chydaeus nigricans* Ito, 2002. However, according to our data, *Chydaeus oblongulus* has never been described, and therefore is a *nomen nudum*. Ito probably was referring to *Chydaeus nigricans* when he listed *Chydaeus oblongulus* because the characters he mentioned (Ito 2003) for *Chydaeus oblongulus* and his reference to his 2002 paper, in which he described *Chydaeus nigricans*, are consistent with this assumption. *Chydaeus nigricans* was treated as a junior synonym of *Chydaeus salvazae* by Kataev and Schmidt (2006).

### Chydaeus bedeli

(Tschitschérine, 1897)

http://species-id.net/wiki/Chydaeus_bedeli

#### Remarks.

*Chydaeus bedeli* is widely distributed over the Himalaya, and in the mountains of western China and northern Indochina. According to Kataev and Schmidt (2002), this species belongs to the *bedeli* group and is represented by five subspecies: the nominotypic subspecies (in the mountains of Sichuan), *Chydaeus bedeli difficilis* Kataev & Schmidt, 2002 (in northeastern Yunnan), *Chydaeus bedeli interjectus* Kataev & Schmidt, 2002 (in the East Himalaya), *Chydaeus bedeli longipennis* Kataev & Schmidt, 2002 (in the Western and Central Himalaya), and *Chydaeus bedeli vietnamensis* Kataev & Schmidt, 2002 (in northern Vietnam).

### Chydaeus bedeli difficilis

Kataev & Schmidt, 2002

http://species-id.net/wiki/Chydaeus_bedeli_difficilis

Figs 62 67 72–73

#### Material examined.

 A total of 341 specimens (184 males and 157 females, including 89 males and 76 females in CAS, 88 males and 78 females in IOZ, and 7 males and 3 females in ZIN and cBL&KB) were examined from the following localities: **China**. **Xizang Autonomous Region**. *Medong County*: 1 female, Medog, Baibung, E Doxong Pass, under rocks along trail, 29.49009°N, 94.95566°E, 3100–4010 m, 15–20.VIII.2005, Huang Hao leg. (IOZ); 1 female, Medog, 1900 m, 27.XI.1998 (IOZ).**Yunnan Province**. *Fugong County*: 19 males, 16 females, Lumadeng, Laoshibali, roadside, 27.07978°N, 98.77328°E, 2305 m, 15.VIII.2005, D. Kavanaugh & D.Z. Dong leg. (CAS, IOZ); 5 males, 3 females, same data, but 27.07831°N, 98.77416°E, 2305 m, 15.VIII.2005, H.B. Liang & J.F. Zhang leg. (CAS, IOZ); 1 female, Lishadi Town, Shibali, around hotel, 27.16536°N, 98.78003°E, 2535 m, 18.VIII.2005, D. Kavanaugh & P. Paquin leg. (CAS); 3 males, 3 females, same data, but 5–6.VIII.2005, D. Kavanaugh leg. (CAS, IOZ, ZIN); 3 males, 1 female, same data, but 27.16530°N, 98.77980°E, 2530 m, 4.VIII.2005, H.B. Liang leg. (CAS, IOZ); 6 males, 5 females, Lishadi Town, 2.8 km W Shibali, road, 27.17405°N, 98.76722°E, 2750 m, 9.VIII.2005, D.Z. Dong leg. (CAS, IOZ); 1 male, 1 female, Lishadi Town, 7 km SW Shibali, river, 27.10220°N, 98.73107°E, 2800 m, 13.VIII.2005, D. Kavanaugh & P. Raquin leg. (CAS); 1 male, 1female, Lumadeng, 8.4 km W Shibali, roadside, 27.18740°N, 98.71936°E, 3160 m, 14.VIII.2005, D. Kavanaugh & D.Z. Dong leg. (CAS); 1 male, 1 female, Lishadi Town, 0.5 km below Shibali, 27.16520°N, 98.77980°E, 2530 m, 5.VIII.2005, H.B. Liang & G. Tang leg. (IOZ); 2 females, Lishadi Town, 0.5 km W Shibali, 27.20192°N, 98.71371°E, 3250 m, 7.VIII.2005, P. Paquin leg. (CAS); 2 females, Lishadi Town, 4 km W Shibali, roadside, 27.17740°N, 98.75490°E, 2800 m, 16.VIII.2005, D.Z. Dong leg. (CAS, IOZ); 1 male, Lishadi Town, 2 km E Shibali, roadside, 27.16100°N, 98.79370°E, 2300 m, 18.VIII.2005, D.Z. Dong leg. (IOZ); 1 male, Lishadi Town, 8.5 km up Shibali, river, 27.18408°N, 98.71882°E, 3095 m, 8.VIII.2005, H.B. Liang & J.F. Zhang leg. (IOZ); 1 male, Lishadi, 9.5 km up Shibali, road, 27.19436°N, 98.71487°E, 3195 m, 14.VIII.2005, H.B. Liang leg. (IOZ); 6 males, 4 females, Lishadi Town, 10 km up Shibali, road, 27.19980°N, 98.71375°E, 3200 m, 16.VIII.2005, J. Zhang leg. (CAS, IOZ); 1 female, same data, but 12.VIII.2005, H.B. Liang & J.F. Zhang leg. (IOZ); 1 male, same data, but 12.VIII.2005, H.B. Liang & J.F. Zhang leg. (IOZ); 2 males, 1 female, Lishadi Town, 6 km up, roadside, 27.17628°N, 98.74167°E, 2920 m, 2.V.2004, H.B. Liang & X.Y. Li leg. (CAS, IOZ); 1 male, 1 female, Lishadi Town, Shibali, 1 km up, roadside, 27.17084°N, 98.76983°E, 2687 m, 1.V.2004, H.B. Liang leg. (IOZ); 2 males, 4 females, Lishadi Town, Shibali, 2 km up, roadside, 27.17156°N, 98.77098°E, 2733 m, 1.V.2004, H.B. Liang leg. (CAS, IOZ); 1 female, Lishadi Town, Shibali, 4 km up, roadside, 27.17750°N, 98.75508°E, 2820 m, 3.V.2004, H.B. Liang & M. Xie leg. (IOZ); 1 male, 3 females, Lishadi Town, Shibali, 12 km up, roadside, 27.20654°N, 98.71772°E, 3280 m, 8.V.2004, H.B. Liang & B.-X. Zhu leg. (CAS, IOZ); 2 males, 2 females, Lishadi Town, Shibali, 10 km up, roadside, 27.19980°N, 98.71375°E, 3200 m, 6.V.2004, day, H.B. Liang & B.X. Zhu leg. (CAS, IOZ, ZIN); 4 males, 1 female, Lishadi Town, Shibali, 1.5 km down road, 27.16284°N, 98.78989°E, 2420 m, 2.V.2004, H.B. Liang & G.X. Peng leg. (CAS, IOZ); 4 males, 5 females, Lishadi Town, 4.3 km above Shibali on Yaping road, 27.17262°N, 98.76943°E, 2826 m, 3.V.2004, D. Kavanaugh leg. (IOZ); 1 male, 1 female, Lumadeng, Yaping-Shibali, roadside, 27.14627°N, 98.81559°E, 2030 m, 3.V.2004, H.B. Liang, M. Xie leg. (IOZ, CAS); 1 female, Lumadeng, Yaping, Rimalige, road, 27.09728°N, 98.80475°E, 2040 m, 4.V.2004, H.B. Liang & B.-X. Zhu leg. (IOZ); 6 males, 6 females, Lumadeng, Yaping, Yejiadi, roadside, 27.08004°N, 98.77325°E, 2307 m, 10.V.2004, H.B. Liang & B.X. Zhu leg. (CAS, IOZ); 3 males, 5 females, Lumadeng, Laoshibali, riverside, 27.07831°N, 98.77416°E, 2305 m, 21.VIII.2005, H.B. Liang & J.F. Zhang leg. (CAS, IOZ); 1 female, Lumadeng, 8 km up Laoshibali, road, 27.10421°N, 98.73274°E, 2800 m, 13.VIII.2005, H.B. Liang & J.F. Zhang (IOZ); 1 male, Fugong (IN BURMA side), Lumadeng, Laoshibali Yakou, road, 27.06427°N, 98.75129°E, 3267 m, 13.VIII.2005, J.F. Zhang leg. (IOZ); 5 males, 10 females, Lumadeng, Laoshibali Yakou, 27.06429°N, 98.75123°E, 3270 m, 13.VIII.2005, D. Kavanaugh & D.Z. Dong leg. (CAS, IOZ); 3 males, 1 female, Maji Town, Majimi Vill., riverside, 27.39630°N, 98.81701°E, 1567 m, 28.IV.2004, H.B. Liang leg. (IOZ); 1 female, Shangpa, Nujiang River, roadside, 27.06428°N, 98.75105°E, 3276 m, 6.V.2004, X. Li leg. (IOZ); 2 males, W Yunnan Province, SW Weideng, 27°00'09"N, 99°00'47"E, 3145 m, 4.VI.2006, I. Belousov & I. Kabak leg. (cBL&KB, ZIN); 2 males, 1 female, W Yunnan Province, NE Fugong, 26°56'46"N, 98°56'13"E - 26°57'32"N, 98°56'44"E, 3240–3449 m, 31.V.2006, 29.V.2006, I. Belousov & I. Kabak leg. (cBL&KB, ZIN). *Gongshan County*: 14 males, 7 females, Cikai Town, Dabadi, riverside, 27°47'48"N, 98°30'21"E, 3000 m, 1.X.2002, X. Li leg. (CAS, IOZ, ZIN); 1 male, Cikai Town, Heipu Yakou to Dahaituo, 27.78440°N, 98.46038°E, 3342 m, 13.VIII.2006, Y. Liu leg. (ZIN); 4 males, 2 females, Cikai Township, 41 km W of Gongshan on Dulong Valley Road at Dabadi, 3000 m, 27.79655°N, 98.50562°E, 27.IX.–6.X.2002, Stop DHK-2002–031A, D. Kavanaugh, P. Marek, H.B. Liang & D.Z. Dong leg. (CAS, IOZ); 2 females, Cikai Township, 8.3–13.1 km to NW of Gongshan on Dulong Valley Road, 2620–3000 m, 27.75653°N, 98.58214°E to 27.78982°N, 98.52802°E, 23.IX.2002, D. Kavanaugh, P. Marek, H.B. Liang & D.Z. Dong leg. (CAS, IOZ); 1 male, Cikai Town, 16.8 km W of Gongshan on Dulong Valley Road at Heiwadi, 2150 m, 27.79584°N, 98.58443°E, 10.X.2002, D. Kavanaugh, P. Marek, H.B. Liang & D.Z. Dong leg. (IOZ); 1 female, Dulongjiang, Moqiewang He, 27.91040°N, 98.41076°E, 2185 m, 8.XI.2004, D. Kavanaugh & M. Dixon leg. (IOZ); 1 male, Bingzhongluo, Gongdangshen Shan, 27°59'51"N, 98°37'7"E, 2540 m, 17.IV.2002, H.B. Liang, W. Ba, G. Yang & X.Q. Li leg. (IOZ); 7 males, 1 females, same data, but 2480 m, 24.IV.2002 (IOZ); 15 males, 14 females, No 12, Bridge to Dulongjiang, 27°42'54"N, 98°30'8"E, 2770 m, 30.IV.-3.V.2002, H.B. Liang & W. Ba leg. (CAS, IOZ); 1 female, Yeniugu, along road, 27°43'3"N, 98°44'14"E, 2020 m, 16.IV.2002, H.B. Liang & W. Ba leg. (IOZ); 3 males, 1 female, Heiwadi, on new road to Dulongjiang, 27°47'39"N, 98°35'13"E, 2020 m, 20.IV.2002, H.B. Liang & W. Ba leg. (IOZ); 2 males, Danzhu, along road, 27°37'50"N, 98°37'14"E, 2600 m, 14.IV.2002, H.B. Liang & W. Ba leg. (IOZ); 18 males, 18 females), Dongshaofang-Yakou, 27°41'40"N, 98°28'47"E, 3400 m, 1.V.2002, H.B. Liang, W. Ba, G. G. Yang & Z.Q. Li leg. (IOZ, CAS); 2 females, Dulongjiang Yakou, 27°46'51"N, 98°28'11"E, 2350 m, 1.X.2002, H.B. Liang leg. (IOZ); 1 female, Danzhu He, 13.5–13.8 km SSW of Gongshan, 2720–2840 m, 27.63267°N, 98.60861°E to 27.63331°N, 98.60356°E, 30.VI.–5.VII.2000, D. Kavanaugh et al. (CAS); 5 males, 3 females, Nujiang State Nature Reserve, Dong Shao Fang area, 18–20 km W of Gongshan, 27.69504°N, 98.48433°E, 3230–3300 m, 16–17.VII.2000, D. Kavanaugh et al (CAS, IOZ); 1 female, Nujiang Pref., Dulong Co., Dulong Jiang, 2 km N of Bapo, 31.4 km W of Gongshan, 1510 m, 27.76000°N, 98.34611°E, 16–17.VII.2000, P. Tomas leg. (IOZ). *Longyang County*: 1 female, Bawan, Nankang station, 24.83178°N, 98.76472°E, 2180 m, 26.V.2005, D.Z. Dong leg. (IOZ); 1 female, same data, but 22.V.2005, D. Kavanaugh & D.Z. Dong leg. (CAS); 5 males, 2 females, same data, but 24.82587°N, 98.76832°E, 2048 m, 22.V.2005, H.B. Liang leg. (CAS, IOZ); 2 males, Bawan, Sanchahe, 24.94849°N, 98.75699°E, 2300 m, 3.VI.2005, D. Kavanaugh & D.Z. Dong leg. (CAS); 1 male, same data, but 2325 m (IOZ); 2 males, 2 females, same data, but 24.94755°N, 98.75564°E, 2300 m, 3.VI.2005, H.B. Liang & H.M. Yan leg. (CAS, IOZ). *Lushui County*: 1 male, 1 female, Pianma, 6 km ESE Pianma, river, 26.00703°N, 98.16209°E, 2254 m, 15.V.2005, D.Z. Dong leg. (IOZ); 2 males, Pianma, Fengxue Yakou, roadside, 25.97288°N, 98.68336°E, 3150 m, 11.V.2005, D. Kavanaugh leg. (CAS); 2 females, same data, but 25.97347°N, 98.68780°E, 3130 m, 17.V.2005 (CAS); 2 males, same data, but 25.97410°N, 98.67716°E, 3120 m, 18.V.2005, D. Kavanaugh & D.Z. Dong leg. (CAS, IOZ); 1 male, Pianma, Changyanhe, riverside, 25.99414°N, 98.66336°E, 2540 m, 12.V.2005, D. Kavanaugh & D.Z. Dong leg. (CAS); 2 males, 1 female, Pianma Yakou, 58.1 km W of Nu Jiang Road on Pianma Road, 3140 m, 25.97288°N, 98.68336°E, 15.X.2002, D. Kavanaugh, P. Marek & H.B. Liang leg. (CAS, IOZ); 6 males, 4 females, Luzhang, Yaojiaping, riverside, 25.97722°N, 98.71091°E, 2527 m, 19.V.2005, D. Kavanaugh & D.Z. Dong leg. (CAS, IOZ); 4 males, 2 females, same data, but 20.V.2003 (CAS, IOZ). *Tengchong County*: 1 female, Jietou, Datang, Dahelingganjiao, 25.7394°N, 98.69630°E, 2010 m, 14.V.2006, D.Z, Dong & X.P. Wang leg. (IOZ); 3 males, same data, but 16.V.2006, D. Kavanaugh leg. (CAS, IOZ); 1 female, same data, but 15.V.2006, H.B. Liang leg. (IOZ); 2 males, 1 female, Mingguang, No. 8 Boundary Post, 25.80984°N, 98.62084°E, 2887 m, 23.V.2006, D. Kavanaugh leg. (CAS, IOZ).

#### Distribution.

**Figures 62–63.**
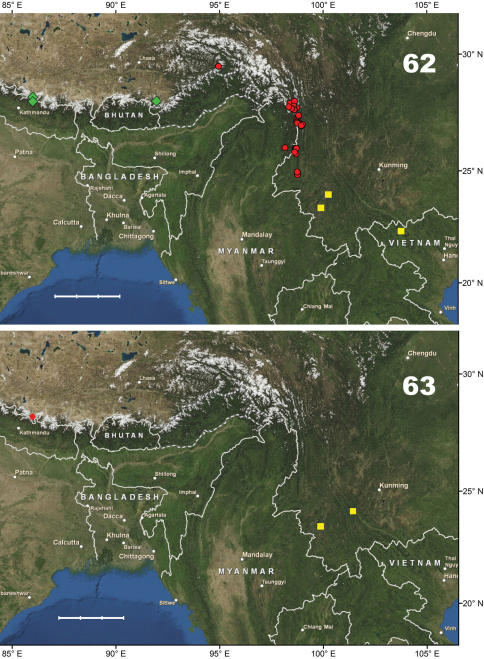
Toporelief map of southeastern Asia, illustrating localities for *Chydaeus* species **62** *Chydaeus bedeli* (Tschitschérine): *Chydaeus bedeli difficilis* Kataev & Schmidt = red dots, *Chydaeus bedeli interjectus* Kataev & Schmidt = green diamonds, and *Chydaeus bedeli vietnamensis* Kataev & Schmidt = yellow squares **63** *Chydaeus similis* Kataev & Schmidt = yellow squares and *Chydaeus irvinei* (Andrewes) = red dot. Only new records reported here are shown. Scale bar = 300 km (in 100 km increments).

Fig. 62. Subspecies *Chydaeus bedeli difficilis* was known previously from only the type series, which was collected in the Hengduan Shan (Mountains) in northwestern Yunnan Province, China (Kataev and Schmidt 2002). Based on the new records presented here,it occurs also in southeastern Xizang Autonomous Region (Medog County) and western Yunnan (in the Gaoligong Shan and Nu Shan mountains in Fugong, Gongshan, Longyang, Lushui, and Tengchong counties).

#### Habitat.

 Specimens were collected in roadside and road cut open areas (Figs 67, 72), on open, disturbed stream banks, and in other disturbed areas (Fig. 73), hidden under stones and other debris during daylight hours and active on the soil surface at night.

**Figures 64–65.**
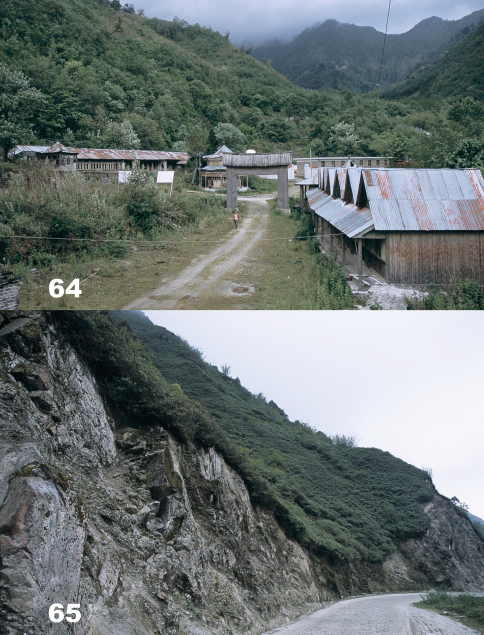
Digital photographs of habitats for *Chydaeus* species **64** Yaojiaping, Lushui County, Yunnan Province, China, ca. 2500 m; locality for *Chydaeus satoi* Ito and *Chydaeus shunichii* Ito **65** Fengxue Yakou, Lushui County, Yunnan Province, China, ca. 3150 m; locality for *Chydaeus bedeli difficilis* Kataev & Schmidt and *Chydaeus shunichii* Ito.

**Figures 66–67.**
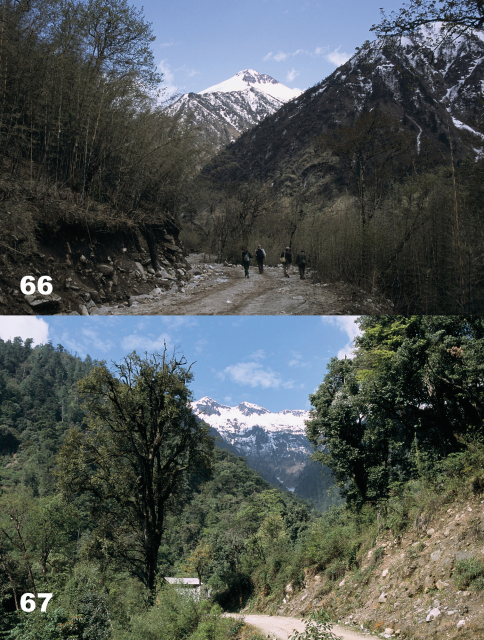
Digital photographs of habitats for *Chydaeus* species **66** Shibali (6 km W), Fugong County, Yunnan Province, China, ca 2920 m; locality for *Chydaeus fugongensis* sp. n. **67** Shibali, Fugong County, Yunnan Province, China, ca. 2530 m; locality for *Chydaeus asetosus* sp. n. and *Chydaeus bedeli difficilis* Kataev & Schmidt

**Figures 68–69.**
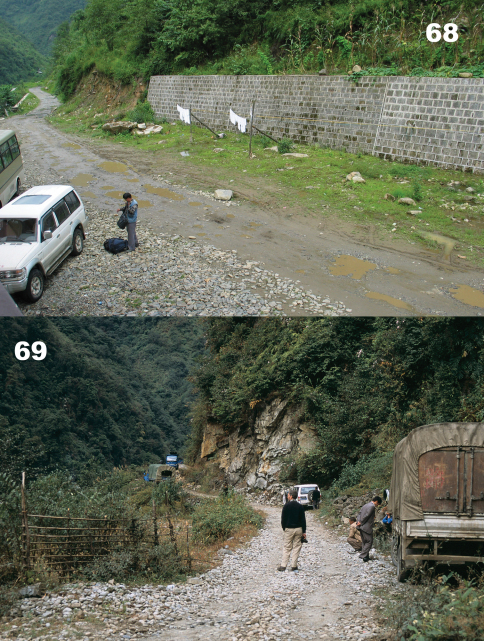
Digital photographs of habitats for *Chydaeus* species **68** Kongdang, Gongshan County, Yunnan Province, China, ca. 1525 m; locality for *Chydaeus andrewsi andrewsi* Schauberger **69** Dulong Valley N of Kongdang, Gongshan County, Yunnan Province, China, ca. 1550 m; locality for *Chydaeus andrewsi andrewsi* Schauberger and *Chydaeus obtusicollis* Schauberger.

**Figures 70–71.**
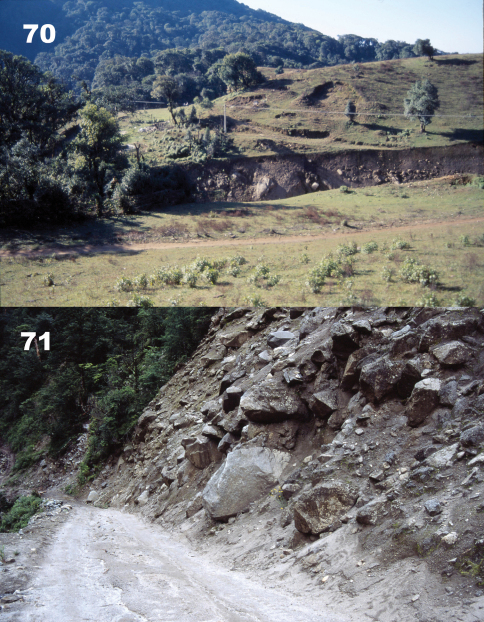
Digital photographs of habitats for *Chydaeus* species **70** Nankang Yakou, Longyang County, Yunnan Province, China, ca. 2130 m; locality for *Chydaeus andrewsi andrewsi* Schauberger, *Chydaeus convexus* Ito, and *Chydaeus salvazae* Schauberger **71** Danzhu Valley, Gongshan County, Yunnan Province, China, ca. 2700 m; locality for *Chydaeus semenowi* (Tschitschérine).

**Figures 72–73.**
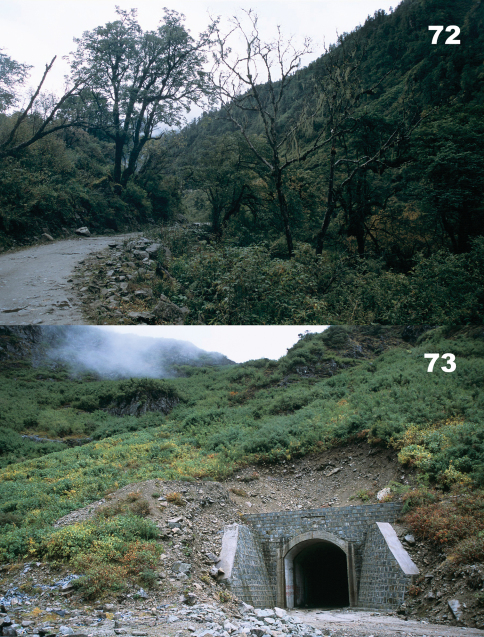
Digital photographs of habitats for *Chydaeus* species **72** Gongshan-Dulongjiang road at Dabadi, Gongshan County, Yunnan Province, China, ca. 2470 m; locality for *Chydaeus andrewsi andrewsi* Schauberger and *Chydaeus bedeli difficilis* Kataev & Schmidt **73** Heipu Yakou, Gongshan County, Yunnan Province, China, ca. 3340 m; locality for *Chydaeus bedeli difficilis* Kataev & Schmidt.

**Figure 74.**
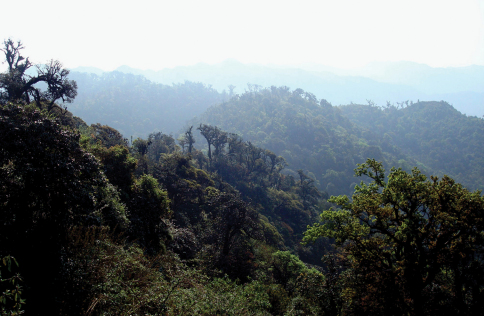
Digital photograph of landscape at Fenshuiling, Jinping County, Yunnan Province, China, ca. 2060 m; locality for *Chydaeus andrewsi kumei* Ito.

### Chydaeus bedeli interjectus

Kataev & Schmidt, 2002

http://species-id.net/wiki/Chydaeus_bedeli_interjectus

Fig. 62

#### Material examined.

 A total of 61 specimens (40 males and 21 females in IOZ) were examined from the following localities: **China**. **Xizang Autonomous Region**. *Cona County*: 2 males, Mama, 2900 m, 6.VIII.1974 (IOZ). *Nyalam County*: 8 males and 7 females), Nyalam, 3900 m, 13.V.1974 (IOZ); 1 male, Nyalam, Zham, 2650 m, 15.V.1966, Wang Shuyong leg. (IOZ); 8 males, 3 females, Nyalam, Zham, Quxam, 3300 m, 8.VII.1975, Huang Fusheng leg. (IOZ); 1 male, same data, but 2250 m, 17.V.1974, Wang Shuyong leg. (IOZ); 1 female, same data, but 3500 m, 21.V.1966, Wang Shuyong leg. (IOZ); 11 males, 2 females, same data, but 3300 m, 7 and 8.VII.1975 (IOZ); 3 males, 1 female, same data, but 3400 m, 7.VII.1975 (IOZ); 3 males, 1 female, same data, but 6.VII.1975 (IOZ); 1 male, same data, but 3500 m, 21.V.1966, Wang Shuyong leg. (IOZ); 1 female, same data, but 2400–3000 m, 11.V.1966, Wang Shuyong leg. (IOZ); 1 female, same data, but 3370 m, 22.V.1966, Wang Shuyong leg. (IOZ). *Yadong County*: 1 male, Tibet, Yadong, 30.V.1975 (IOZ); 1 female, same data, but 31.V.1975 (IOZ); 2 female, same data, but 1.VIII.1981 (IOZ); 1 male, same data, but 2800 m, 30.V.1975 (IOZ); 1 male, same data, but 2800 m, 31.V.1974 (IOZ); 1 female, same data, but 2800 m, 5.VI.1961 (IOZ).

#### Distribution.

Fig. 62. *Chydaeus bedeli interjectus* is common in the Eastern Himalaya, from the eastern part of Nepal to Bhutan (Kataev and Schmidt 2002). This taxon was not previously recorded from China; and new records presented here extend its known range to include the southern part Xizang Autonomous Region (Cona, Nyalam, and Yadong counties), at elevations of 2250-3900 m.

### Chydaeus bedeli vietnamensis

Kataev & Schmidt, 2002

http://species-id.net/wiki/Chydaeus_bedeli_vietnamensis

Fig. 62

#### Material examined.

A total of 6 specimens (4 males and 2 females, including 4 males and 1 female in cBL&KB and ZIN and 1 male in cFED) were examined from the following localities: **China**. **Yunnan.** *Longxiang County (District)*: 1 male, ENE Lincang, 23°57'36"N, 100°15'21"E, 3340 m, 28.VI.2011, I. Belousov, I. Kabak & A. Korolev leg. (ZIN). *Shuangjiang County*: 3 males, SSE Shuangjiang Town, 23°22'32"N, 99°53'47"E – 23°22'22"N, 99°53'26"E, 2790-2950 m, 23.VI.2011, I. Belousov, I. Kabak & A. Korolev leg. (cBL&KB, ZIN). **Vietnam.** **Lao Cai Province.** 1 female, 6 km W of Sa Pa, N slope of Phansipan Mt. Area, 2000–2100 m, near Tram don (base of Hoang Lien Nat. Park), 22°21'N, 103°46'E, V.2005, A.V. Abramov leg. (Exp. of Russia-Vietnam Tropical Centre) (ZIN); 1 male, Sa Pa env., ca 1600 m, V.2006, A. Anitchkin leg. (cFED).

#### Distribution.

 Previously, *Chydaeus bedeli vietnamensis* was known from a single male collected in northern Vietnam (type locality: northern slope of Fansipan Mt., Sa Pa, 22°17'N, 103°44'E, 1525 m). Based on the new records reported here, this subspecies occurs also in southern Yunnan Province, China (Fig. 62).

#### Remarks.

 Specimens from Yunnan are similar in external features and male genitalia to those from Vietnam, but they are slightly smaller (body length 9.0-10.2 mm versus 10.7–10.8 mm in males from Vietnam, including the holotype) and have more markedly prominent denticles at their basal pronotal angles. We add the following mensural data to describe the Yunnan specimens and thereby expand on Kataev & Schmidt’s description of this taxon: Proportions (males from Yunnan): HWmax/PWmax = 0.71–0.72; HWmin/PWmax = 0.59–0.61; PWmax/PL = 1.52–1.59; PWmax/PWmin = 1.26–1.37; EL/EW = 1.34–1.44, EL/PL = 2.62–2.85, EW/PWmax = 1.23–1.28.

### Chydaeus similis

Kataev & Schmidt, 2002

http://species-id.net/wiki/Chydaeus_similis

Fig. 63

#### Material examined.

A total of 19 specimens (15 males and 4 females in cBL&KB and ZIN) were examined from the following localities: **China. Yunnan:** *Xinping County*: 1 female, Ailaoshan Mt. Range, W Shuitangzhen Town, 24°08'24"N, 101°25'16"E, 1940 m, 2.VI.2011, I. Belousov, I. Kabak & A. Korolev leg. (cBL&KB); 15 males, 3 females, SSE Shuangjiang Town, 23°22'22"N, 99°54'47"E, 2540 m, 22.VI.2011, I. Belousov, I. Kabak & A. Korolev leg. (cBL&KB, ZIN).

#### Distribution.

 The geographical range of *Chydaeus similis* in China extends from Central Sichuan to Yunnan. In Yunnan, it had been recorded previously only from the Gang Shan (Mountains) (Kataev and Schmidt 2002). Based on the new records reported here (Fig. 63), *Chydaeus similis* is more widely distributed in Yunnan.

#### Remarks.

 Like the preceding species, *Chydaeus similis* belongs to the *bedeli* group (Kataev and Schmidt 2002). In specimens of *Chydaeus similis* from Yunnan, the denticles at the pronotal basal angles are small and only slightly prominent, smaller in most specimens than in Yunnan specimens of *Chydaeus bedeli vietnamensis*, members of which are very similar in habitus to *Chydaeus similis* and occur in the same area.

### Chydaeus irvinei

(Andrewes, 1930)

http://species-id.net/wiki/Chydaeus_irvinei

Fig. 63

#### Material examined.

 A total of 5 specimens (3 males and 2 females, all in IOZ) were examined from the following locality: **China**. **Xizang Autonomous Region**. *Nyalam County*: 2 males, 28°10'N, 85°57'E, 3300 m, 17.V.1966, Wang Shuyong leg.; 1 female, same data, but 3570 m, 18.V.1966; 1 female, same data, but 2400–3400 m, 11.V.1966; 1 male, same data, but 3400 m, 6.VIII.1971, Zhang Xuezhong leg.

#### Distribution.

 This species, a member of the *irvinei* group, was known previously from two isolated areas in the Central Himalaya: to the north of Sikkim in southern Xizang and in the upper Tama Koshi valley in southern Xizang and Central Nepal (Kataev and Schmidt 2002). The new record reported here (Fig. 63) is in the upper Bhote Koshi valley in southern Xizang, not far to the west from the Tama Koshi valley.

## Supplementary Material

XML Treatment for Chydaeus shunichii

XML Treatment for Chydaeus fugongensis

XML Treatment for Chydaeus gutangensis

XML Treatment for Chydaeus hanmiensis

XML Treatment for Chydaeus asetosus

XML Treatment for Chydaeus baoshanensis

XML Treatment for Chydaeus satoi

XML Treatment for Chydaeus obtusicollis

XML Treatment for Chydaeus convexus

XML Treatment for Chydaeus malaisei

XML Treatment for Chydaeus semenowi

XML Treatment for Chydaeus andrewesi

XML Treatment for Chydaeus andrewesi andrewesi

XML Treatment for Chydaeus andrewesi kumei

XML Treatment for Chydaeus salvazae

XML Treatment for Chydaeus bedeli

XML Treatment for Chydaeus bedeli difficilis

XML Treatment for Chydaeus bedeli interjectus

XML Treatment for Chydaeus bedeli vietnamensis

XML Treatment for Chydaeus similis

XML Treatment for Chydaeus irvinei
